# Mapping socio-economic status using mixed data: a hierarchical Bayesian approach

**DOI:** 10.1093/jrsssa/qnae080

**Published:** 2024-08-20

**Authors:** Gabrielle Virgili-Gervais, Alexandra M. Schmidt, Honor Bixby, Alicia Cavanaugh, George Owusu, Samuel Agyei-Mensah, Brian Robinson, Jill Baumgartner

**Affiliations:** 1 Department of Epidemiology, Biostatistics and Occupational Health, McGill University, Montreal, QC, Canada; 2 Institute of Public Health and Wellbeing, University of Essex, Colchester, UK; 3 Department of Geography, McGill University, Montreal, QC, Canada; 4 Institute of Statistical, Social & Economic Research, University of Ghana, Legon-Accra, Ghana; 5 Department of Geography and Resource Development, University of Ghana, Legon-Accra, Ghana; 6 Department of Equity, Ethics, and Policy, McGill University, Montreal, QC, Canada

**Keywords:** Bayesian hierarchical modelling, conditional auto-regressive models, factor analysis, greater Accra metropolitan area, socio-economic status

## Abstract

We propose a Bayesian hierarchical model to estimate a socio-economic status (SES) index based on mixed dichotomous and continuous variables. In particular, we extend Quinn’s ([2004]. Bayesian factor analysis for mixed ordinal and continuous responses. *Political Analysis, 12*(4), 338–353. https://doi.org/10.1093/pan/mph022) and Schliep and Hoeting’s ([2013]. Multilevel latent Gaussian process model for mixed discrete and continuous multivariate response data. *Journal of Agricultural, Biological, and Environmental Statistics, 18*(4), 492–513. https://doi.org/10.1007/s13253-013-0136-z) factor analysis models for mixed dichotomous and continuous variables by allowing a spatial hierarchical structure of key parameters of the model. Unlike most SES assessment models proposed in the literature, the hierarchical nature of this model enables the use of census observations at the household level without needing to aggregate any information *a priori*. Therefore, it better accommodates the variability of the SES between census tracts and the number of households per area. The proposed model is used in the estimation of a socio-economic index using 10% of the 2010 Ghana census in the Greater Accra Metropolitan area. Out of the 20 observed variables, the number of people per room, access to water piping and flushable toilets differentiated high and low SES areas the best.

## 1 Motivating example: the greater Accra metropolitan area

In order to glimpse socio-economic realities, authors have turned to model-based data reduction techniques to estimate indices capable of capturing such a complex concept. Most rely on strictly continuous variables and summarize information from small to large areas. Common practices involve factor analysis, weighted averages and principal component analysis (PCA). This paper proposes a hierarchical model that extends factor analysis using mixed continuous and dichotomous variables and a novel application to socio-economic status (SES) estimation, thereby demonstrating the flexibility of hierarchical models. The proposed model accounts for local heterogeneity by estimating neighbourhood-level indices using individual household-level information and accounting for potential spatial structure present in the data.

In the last decades, Ghana has seen fast urbanization of its population, especially around its capital Accra. According to the World Bank, the Ghanaian urban population has tripled since the 80s, leading to unregulated urban expansion and limited access to quality services and housing (Group, 2015). Because of this fast urbanization, its metropolitan area comprises many social discrepancies, showing groups of wealthy and vulnerable populations. UN-habitat stated those most vulnerable were largely affected by inadequate drinking water, sanitation, and housing structures (UN-Habitat, 2004). To assess quantitatively what drives deprivation in the urban setting, we want to estimate an index capable of summarising the complex realities of SES. As Webb et al. (2017), we define SES as a combination of economic, human and social resources a household or community has access to. Using limited information, we will be able to map areas in need of services and identify key characteristics differentiating low and high-SES neighbourhoods. This statistic aims to simplify to one dimension a multidimensional reality to serve as a tool for local development actors.

We use the publicly available 10% of census data in the greater Accra metropolitan area (GAMA). This sample was formed by the Ghana Statistical Service by systematically selecting every 10th respondent to the census. We did not have access to the full census data, and it is the most recent census publicly available for Ghana. The data includes categorical and continuous variables obtained from households in the census. Each household is located inside an enumeration area (EA), similar to a census tract. In turn, each area is nested in a neighbourhood, part of one of the three districts forming GAMA: La Dade Kotopon, Ledzokuku-Krowor, and Ashiedu Keteke. Information about the location of these EA’s is available, but not the location of households within those EA’s. In total, there are 2,424 areas with 56,231 households within them and counting over 132,000 respondents.

Variables were selected with the aim to describe a household’s living conditions, environment, and available services also their education, access to information, and income level. Given the economic realities of GAMA, we did not have access to household revenue or consumption data. Each variable was coded in such a way that a higher value should imply a *lower SES*. Because some of the retained categorical variables presented low counts in certain areas and categories, they were recoded as dichotomous variables. The dichotomization and selection of variables were done in accordance with the UN’s slum index (UN-Habitat, 2004) and the World Health Organization’s recommendations (Organization, 2016). The findings of Songsore and McGranahan (1993, 1998) who have done extensive work on inequalities in Accra and Moore et al. (2003) were also reviewed. Finally, the selection was approved by Ghanaian scholars and practitioners with expertise in urban and economic development through the Pathways to Healthy Cities initiative (see https://equitablehealthycities.org/). In all, 20 variables were selected, each expressed at the household level, including 5 continuous and 15 dichotomous variables. Each variable and its definition can be found in Section A of the online supplementary material. Only complete data was retained in the study.

When considering aggregated measures, they present high correlations (see Section C of the online supplementary material). This relation is suitable for factor analysis as it supposes this correlation is due to an underlying common factor, here labelled as SES. When we aggregate the variables at the area level, they present varying degrees of spatial patterns. Variables such as illiteracy, and no computer and cellphone ownership seem to cluster in areas known as low SES dwellings, while others like monoparental households and unemployment do not show spatial patterns. Moran’s statistics on area-level variables ranged from 0.02 (monoparental households) to 0.33 (no secondary education) with an average of 0.16. Variables showing the biggest statistics (>0.25) included the ratio of people to room, the proportion of illiteracy, owning no flush toilet, unimproved liquid waste methods and not having access to a toilet (shared or not, flushable or not). The spatial independence assumption was rejected (*p*-values < 0.05) for all variables except the proportion of unemployment, indicating that allowing the latent factor to follow a spatial structure could be suited to the Ghana data. Histograms of the continuous variables were also computed at the area level and suggested that the proportion of illiteracy and unemployment within households could benefit from being logged for continuous variables to be considered normally distributed.

### 1.1 A brief review of data reduction techniques

Data reduction techniques now have an established history of being used to capture social conditions under various terminologies such as asset ownership, material and social deprivation, vulnerability and SES indices. Variables considered often include assets, access to services and education. Many have used PCA to produce weighted averages of proportions or continuous variables (Chan et al., 2015; Filmer & Pritchett, 2001; Gwatkin et al., 2000; Lalloué et al., 2013; Pampalon et al., 2009; Webb et al., 2017). Some used percentile rankings to obtain the variable weights (Biggs et al., 2021; Flanagan et al., 2011) or *Z*-scores (Santana et al., 2015; Townsend, 1987). Others used factor analysis on aggregated measures over areas of interest (Chan et al., 2015).

The goal of these approaches is to reduce to a single dimension the different traits shared by populations most likely to be in vulnerable situations in urban settings. Instead of using multiple indicators, they make use of the correlation structure existing between the variables that could be explained by a single latent construct. In factor analysis, this observed correlation structure is due to the latent factor of interest, which hypothetically underlays every variable in the model. Most previous studies used summary statistics at the census tract level and implicitly assumed that each area contained a similar number of households and that their populations were homogeneous, which might not hold true. Furthermore, they ignored the data’s hierarchical structure, affecting the estimates’ precision. PCA, being an exploratory tool, does not permit direct estimation of the uncertainty of the estimated index. Moreover, PCA and factor analysis do not produce reliable estimates when used on dichotomous variables. Kolenikov and Angeles (2009) proposed the use of polychoric PCA to correct the estimation of the covariance matrix on dichotomous variables. Others like Amek et al. (2015) and Booysen et al. (2008) turned to multiple component analysis to correctly estimate component weights for categorical data at the household level. However, these methods can only accommodate one data type at a time.

To account for the hierarchical nature of the data and its spatial structure, an intuitive approach is to use a hierarchical spatial factor analysis model on continuous aggregated data. For instance, Lopes et al. (2012) measured the vulnerability of the population of Uruguay to vector-borne diseases using a spatial hierarchical model. Hogan and Tchernis (2004) used Townsend’s definition to produce a spatially constructed deprivation index. An alternative to factor analysis when considering non-aggregated dichotomous variables is to use item response theory (IRT). May (2006) applied IRT to estimate an SES index using parental education and home possessions at the house-hold and national levels. Palayew et al. (2021) estimated a deprivation index of HIV/HCV-coinfected individuals. Wang and Wall (2003) generalized hierarchical factor analysis under the Bayesian paradigm to accommodate discrete outcomes using Poisson and binomial models. As factor analysis and IRT present similar model structures and both aim at estimating an underlying construct to correlated variables, Quinn (2004) proposed an alternative combining both methods, allowing the use of ordinal and continuous variables within factor analysis. Pemstein et al. (2010) used it to model a unified democracy score and Clinton and Lewis (2008) to model administrative agencies’ political preferences. Schliep and Hoeting (2013) extended the work of Quinn (2004) to account for an underlying spatial structure of the estimated factor. As their observations varied continuously across the region, they assigned a Gaussian process prior to the latent effect. The model was used to estimate wetland conditions.

This paper proposes a model that uses the information at the household level to estimate an index at the census tract level and identify key attributes of SES, thereby naturally accounting for the possible heterogeneity of within census tract population and of the number of households within them. It combines factor analysis and IRTs, adding hierarchical layers to the model proposed by Quinn (2004). Its hierarchical nature enables the specification of spatially structured prior distributions of key parameters of the model. Schliep and Hoeting (2013) used point-referenced observations and Wang and Wall (2003) incorporated only one observation per area, and their models were applied to a limited amount of data. Our motivating example has a complex, high-dimensional structure that unfolds the full potential of hierarchical mixed modelling. Different from previous references, we propose an extensive simulation study showing how our model can adequately recover the latent parameters. This paper is organized as follows: in the next section, the proposed model and simulation results are presented, Section 3 describes the results of the Ghana case study and Section 4 discusses said results.

## 2 Proposed model

Let *N* be the total number of areas considered and *n*_*i*_ be the number of households within area *i* = 1, 2, …, *N*. Let ***θ*** = (*θ*_1_, …, *θ*_*N*_)^′^, be an *N*-dimensional vector, each component representing the latent factor score associated with area *i*. This score quantifies the socio-economic status of the area. We consider a set of *p* manifest variables, which can be either continuous or dichotomous. Let *y*_*ijk*_ be the *k*th observed variable of the *j*th household in the *i*th EA, where *i* = 1, 2, …, *N, j* = 1, 2, …, *n*_*i*_ and *k* = 1, 2, …, *p*. We consider a latent variable $y_{ijk}^{*}$ such that:

$$y_{ijk}=\left\{ \begin{array}{l} y_{ijk}^{*}\;\text{if}\;\text{the}\;\text{variable}\;k\;\text{is}\;\text{continuous},\\ 1\;\text{if}\;\text{the}\;\text{variable}\;k\;\text{is}\;\text{dichotomous}\;\text{and}\;y_{ijk}^{*}\geq 0,\\ 0\;\text{otherwise.}\; \end{array}\right.$$

Then, assume that the latent variable is described as:

(1) $$y_{ijk}^{*}=\alpha_{ik}+\beta_{k}\theta_{i}+\epsilon_{ijk}.$$

The latent scores, elements of ***θ***, represent the index of interest which summarises the correlation structure of the manifest variables at the area level. The random errors, *ϵ*_*ijk*_, are assumed independent across observations and EAs; they follow a zero mean normal distribution with unknown standard deviation *σ*_*k*_. The error structures of the variables depend on their dichotomous or continuous natures. To clarify these structures, the following paragraphs describe the model for both scenarios.

### The case of continuous variables

We assume that *y*_*ijk*_ follows a normal distribution with mean *α*_*ik*_ + *β*_*k*_*θ*_*i*_ and standard deviation *σ*_*k*_, that is,

(2) $$y_{ijk}\sim N\left(\alpha_{ik}+\beta_{k}\theta_{i},\sigma_{k}^{2}\right).$$

For such variables, *α*_*ik*_ represents the overall mean of the *k*th variable in the *i*th area while the elements of ***β*** = (*β*_1_, …, *β*_*p*_)′ represent the factor loading, a higher value indicating higher importance of the *k*th variable in determining an area’s index. To simplify model interpretation, the elements of ***α*** = (***α*·**_**1**_^′^, …, ***α*·**_***p***_′)′ corresponding to continuous variables are set to zero and these variables are centered.

### The case of dichotomous variables

We assume that

(3) $$y_{ijk}\sim \mathrm{Bernouilli}\left(\text{Φ}\left(y_{ijk}^{*}\right)\right),$$

where Φ(•) denotes the cumulative distribution function of the standard normal distribution. The interpretation aligns with the IRT literature. The elements of ***α*** can be interpreted as negative item difficulty: it shifts the probability of scoring one for such a variable right and left on a latent index axis. A higher negative difficulty (*α*_*ik*_) implies it is easier to score one for that variable, such that very few households in the area *i* will show zeros. Hence, a wider interval of plausible index scores underlays a value of one for that variable. Similarly, the elements of ***β*** can be interpreted as item discrimination: a higher value of *β*_*k*_ means the *k*th variable plays a key role in differentiating between high and low SES households in every area.

### 2.1 Prior specification

We follow the Bayesian paradigm to estimate the parameters of the model. Because most of the variables considered showed a spatial pattern, we find it reasonable to assume that the underlying factor governing their variation follows a prior spatial structure. Let ***θ*** = (*θ*_1_, …, *θ*_*N*_)′ be the latent index we wish to estimate. We assume a proper conditional autoregressive (CAR) prior for ***θ*** with a conditional precision parameter set to **1**, that is, $\left(\theta_{i}|\theta_{j},i\neq j,{ı}_{\theta }\right)\sim N\left(\frac{l_{\theta }{\text{Σ}}_{i\sim j}\theta_{j}}{d_{i}},\frac{1}{d_{i}}\right)$ where *i* ~ *j* denotes areas *i* and *j* are neighbours, *d*_*i*_ denotes the total number of neighbours of area i and *ι*_*θ*_ ~ *U*(0, 1) is a parameter that captures the strength of the spatial structure among the elements of ***θ***. Therefore, using a proper CAR allows the data to drive the inference and inform the model if the components of ***θ*** are spatially structured. In Section 3, we also fit models that assume *θ*_*i*_ to be independent across areas and identically distributed, following a standard normal distribution, that is, *θ*_*i*_ ~ *N*(0, 1), *a priori*. Note further that both the conditional variance in the proper CAR, and the variance of the independent prior, are fixed at 1. We discuss the reason for this below, in the paragraph about identifiability issues.

Let ***α***_.*k*_ = (*α*_1*k*_, …, *α*_*Nk*_)′ be the vector of the difficulty parameter for the *k*th variable of the *N* areas. The following prior specification applies only to the dichotomous variables of the model because the continuous variables are centered, and thus, their respective *α*_*ik*_ are set to zero. As populations vary across the areas, the effect of each variable could be thought to vary as well. We suppose the values of ***α*** to be random effects such that $\alpha_{ik}\sim N\left(\alpha_{k}^{*},1\right)$ where $\alpha_{k}^{*}\sim N(0,1)$, allowing different difficulties in each area such that *α*_*ik*_ and *α*_*jk*_ are conditionally independent given $\alpha_{k}^{*}$. Furthermore, in Section 3, we also fit models with a simpler structure for ***α*** which assume the difficulty parameters to be constant across areas, such that *α*_*ik*_ = *α*_*k*_ ~ *N*(0, 1) for *i* = 1, …, *N*. Let ***σ*** = (*σ*_1_, …, *σ*_*p*_)′ be the vector of the standard deviations of *ϵ*_*ijk*_, for *k* = 1, 2, …, *p*, we assign a half-Cauchy prior, that is, *σ*_*k*_ ~ Half-Cauchy(0, 1) (Gelman, 2006) for continuous variables and assume it to be fixed at one for dichotomous variables (Quinn, 2004; Schliep & Hoeting, 2013).

### 2.2 Inference procedure

Let ***Y*** = (***Y***_**1**.._, …, ***Y***_***N***.._)^′^ be the vector of observations for each area, such that $Y_{i..}={\left(Y_{i1.,},\ldots ,Y_{in_{i}}\right)}^{\prime }$ contains the observations of each household within the area and ***Y***_***ij***._ = (*y*_*ij*1_, …, *y*_*ijp*_)^′^ in turn contains the manifest variables for these households. Let Y* be the latent variables associated with them. Let ***α*** be a *N* × *p* matrix of item negative difficulties with elements set to 0 for continuous variables, ***β*** = (*β*_1_, …, *β*_*p*_)′ be the vector of item discriminations and factor loadings, ***θ*** = (*θ*_1_, …, *θ*_*N*_)′ be the latent factor scores and **Σ** = *diag*(*σ*_1_, …, *σ*_*p*_) be the standard deviations of the random errors, with elements set to 1 if the *k*th variable is dichotomous. Following the Bayesian paradigm, the posterior distribution is proportional to the likelihood multiplied by the prior distribution, which is given by

$$\begin{array}{l} P\left(Y^{*},\alpha ,\beta ,\theta ,\Sigma |Y\right)\propto P\left(Y|Y^{*}\right)P\left(Y^{*}|\alpha ,\beta ,\theta ,\Sigma \right)P(\alpha )P(\beta )P(\theta )P(\Sigma )\\ \;\propto \{\prod_{k=1}^{p}\prod_{j=1}^{n_{i}}\prod_{i=1}^{N}\{I(\text{is}\;\text{continuous}\;)I\left(y_{ijk}=y_{ijk}^{*}\right)\\ \;+I(\text{is}\;\text{dichotomous}\;)\left(I\left(y_{ijk}=1\right)I\left(y_{ijk}^{*}\geq 0\right)+I\left(y_{ijk}=0\right)I\left(y_{ijk}^{*}\leq 0\right)\right)\}\\ \;P_{N}\left(y_{ijk}^{*}|I(\text{is}\;\text{dichotomous}\;)\alpha_{ik}+\beta_{k}\theta_{i},\sigma_{k}^{2}\right)\}P(\alpha )P(\beta )P(\theta )P(\Sigma ), \end{array}$$

where *I*(*x*) denotes an indicator function which is equal to 1 if *x* is true and 0 otherwise; *P*_*N*_(*x* |*μ, σ*^2^) denotes the probability density function of a normal distribution with mean *μ* and variance *σ*^2^ evaluated at *x*. As the previous equation does not have a closed form, we resort to Markov chain Monte Carlo methods (MCMC) to obtain samples from the resulting posterior distribution. One possible approach is to use the data augmentation algorithm for binary data proposed by Albert and Chib (1993). Here, we opted to use the Hamiltonian Monte Carlo algorithm through the Stan programming language within R (R Core Team, 2020; Stan Development Team, 2018, 2022). This programme has a default no-U-turn sampler (NUTS). NUTS was first introduced by Hoffman and Gelman (2014) as an efficient alternative to Hamiltonian Monte Carlo, a type of MCMC sampler, that does not require the choice of a tuning parameter.

#### Identifiability issues

Some constraints need to be imposed to identify the parameters in the model. First, as in traditional factor analysis, there is no unique solution for ***θ*** or ***β*** as the model is invariant under orthogonal transformations. More specifically, as shown in Geweke and Zhou (1996), Lopes and West (2004) and Wang and Wall (2003), if one multiplies the factor loading by a constant and divide the factor by this same constant *c*, that is if we let $\beta_{k}^{*}=c\beta_{k}$ and $\theta_{i}^{*}=\frac{1}{c}\theta_{i}$ for *c* ≠ 0, then $\beta_{k}^{*}\theta_{i}^{*}=\beta_{k}\theta_{i}$. A common remedy to this identifiability issue is to fix the prior variance of the factor at 1. Following Wang and Wall (2003), note that if we assume that *θ*_*i*_ ~ *N*(0, *τ*^2^), with *τ*^2^ ≠ 1, following equation (1), $E\left(y_{ijk}^{*}\right)=\alpha_{ik}$ and $Var\left(y_{ijk}^{*}\right)=\beta_{k}^{2}\tau^{2}+\sigma_{k}^{2}$. If we let *τ*^2^ = 1, then the variance of $y_{ijk}^{*}$ can be uniquely determined by the factor loading *β*_*k*_ and the variance of the measurement error, $\sigma_{k}^{2}$. Wang and Wall (2003) claim that if *τ*^2^ cannot be identified in the independent setup for *θ*_*i*_, it will not be possible to identify it when a spatial structure is assumed for *θ*_*i*_. Therefore, we fix the prior variance of *θ*_*i*_ at 1, regardless of the prior specification for *θ*_*i*_. This same approach was pursued by, e.g. Quinn (2004), Abellan et al. (2007) and Schliep and Hoeting (2013).

We performed simulation studies to confirm we could not estimate the precision parameter of the proper CAR prior for ***θ***. The same simulations highlighted an indeterminacy when the variances of *α*_*ik*_ were free. We chose to set the variance of all *α*_*ik*_ to one around their respective means $\alpha_{k}^{*}$. This simulation study is briefly discussed in Section 2.3 and extensively in Section D of online supplementary material.

Regarding the factor loadings, *β*_*k*_, they cannot vary with the areas, as their effect will not be disentangled from the effect of ***θ***. A further constraint needs to be imposed on the factor loadings as *β*_*k*_*θ*_*i*_ = (−*β*_*k*_)(−*θ*_*i*_). One remedy is to assign a strictly positive prior to the first factor loading *β*_1_ (Abellan et al., 2007; Geweke & Zhou, 1996; Lopes & West, 2004). In all fitted models, we assumed all *β*_*k*_’s to be positive, and assigned independent Half-Normal prior distributions, that is, *β*_*k*_ ~ half − Normal(0, 1). This is for easier interpretation of the factor loadings (Lopes, 2014; Quinn, 2004). As part of a sensitivity analysis, we fit the model restricting only *β*_1_ to be positive and assigned a standard normal distribution prior to *β*_2_, …, *β*_*k*_. We observed no change in the magnitude of the variables’ effects and only one value to be negative (unimproved liquid waste), though close to zero.

### 2.3 Simulation study

To better assess parameter identification and recovery, we conducted a simulation study. For each scenario considered, we generated 100 data sets. Each dataset was composed of a subset of 200 adjacent neighbourhoods from the Ghana data, which comprised 5,195 observations. All neighbourhoods shared borders with at least another one. For each observation, we generated 15 dichotomous and 5 continuous variables. The true spatial intensity parameter was set to 0.9. In the true underlying model, ***θ*** followed a proper CAR, with a hierarchical structure on the difficulty parameters and variance set to one. The true parameter values were the estimates obtained from fitting the model to the full Ghana data. For each scenario and each simulated dataset, we ran the respective Stan model for 10,000 iterations of one MCMC chain and saved the full samples. Each time, values of *α*_*ik*_ and the observed variables were generated.

**Figure 1:**
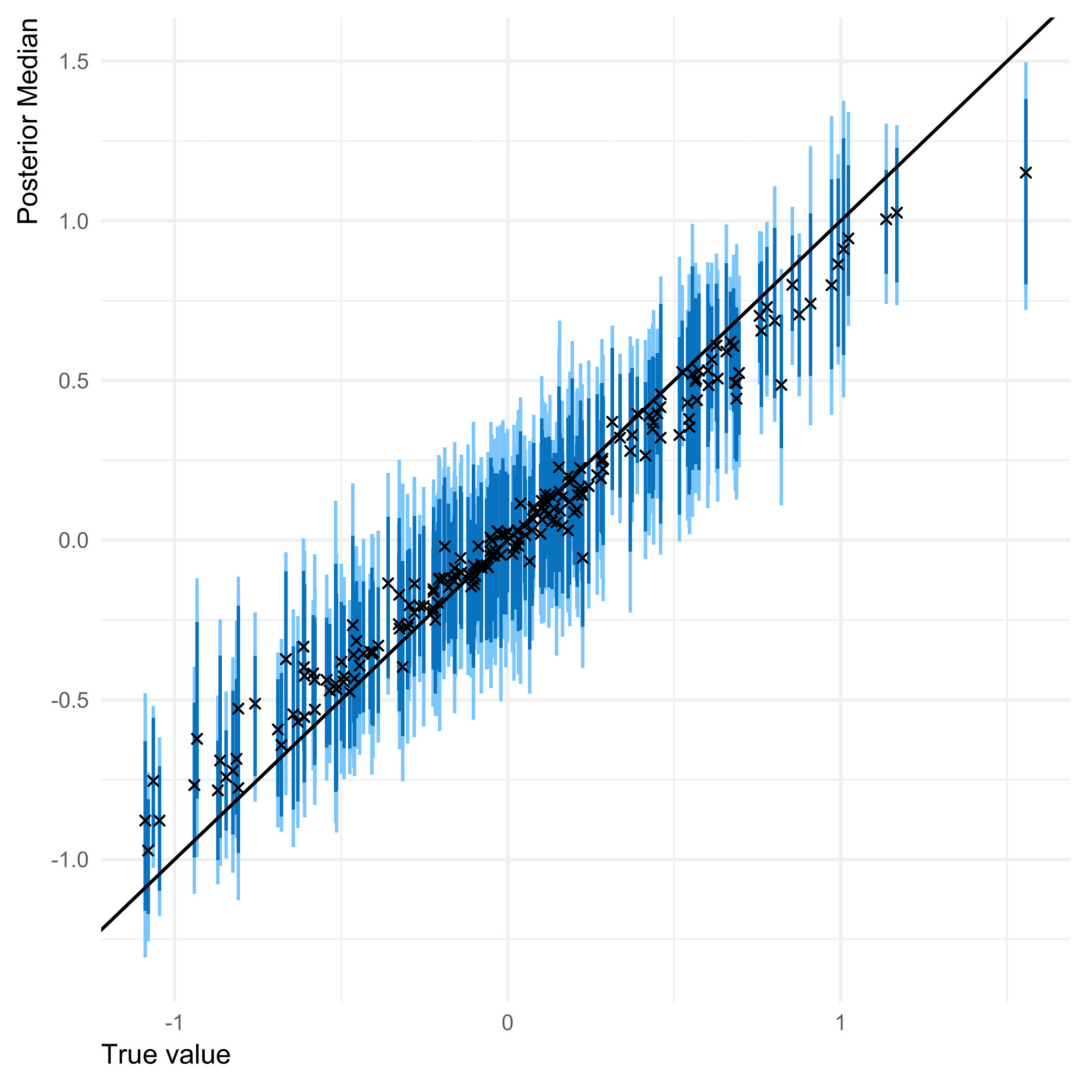
Simulation study results when ***θ*** follows a proper CAR distribution and the variance of the hierarchical difficulties equals one. Light blue segments represent 95% intervals for the median values, while dark blue segments represent 90% intervals. True values are shown as black crosses.

Overall, we can recover all parameters of interest in the model (Figure 1). Credible intervals offer coverage rates close to the expected ones (around 95%) and posterior medians are shown to adequately estimate the parameters. However, variables with low discrimination parameters might be over-estimated and should be interpreted with caution. This simulation study also permitted to observe that *p* free parameters could not be estimated to allow different variances of the hierarchical difficulty. Thus, we concluded, we should fit the model using a fixed variance of one. More details are found in Section D of online supplementary material.

## 3 Estimating SES in Accra, Ghana

We fit particular versions of the proposed model (see Table 1). Each model was run using two MCMC chains for 10,000 iterations. Convergence was assessed using effective sample size (ESS), $\hat{R}$ and trace plots. Models were then compared through the Watanabe-Akaike Information Criterion (WAIC). The WAIC was computed with the log-pointwise predictive density to measure predictive accuracy and expected posterior variance of the log predicted density to correct for the model’s complexity (Gelman et al., 2014). The analysis of Pearson’s residuals did not reveal unusual patterns.

### Results

All the models’ trace plots showed no concerning pattern. All parameters had a value of $\hat{R}$ less than 1.01 and an ESS of at least 10,000, suggesting that the MCMC chains have converged. See Section E.1 of online supplementary material for trace plots of the chains. Using WAIC, the best-fitted model is the main hierarchical intercept model with a spatial prior on the SES index (HS), see Table 2 for details. This model also presents a great gain in the precision of the estimates when comparing the posterior summaries of the SES index of all four models, as seen in Section E.2 of online supplementary material.

**Figure 2:**
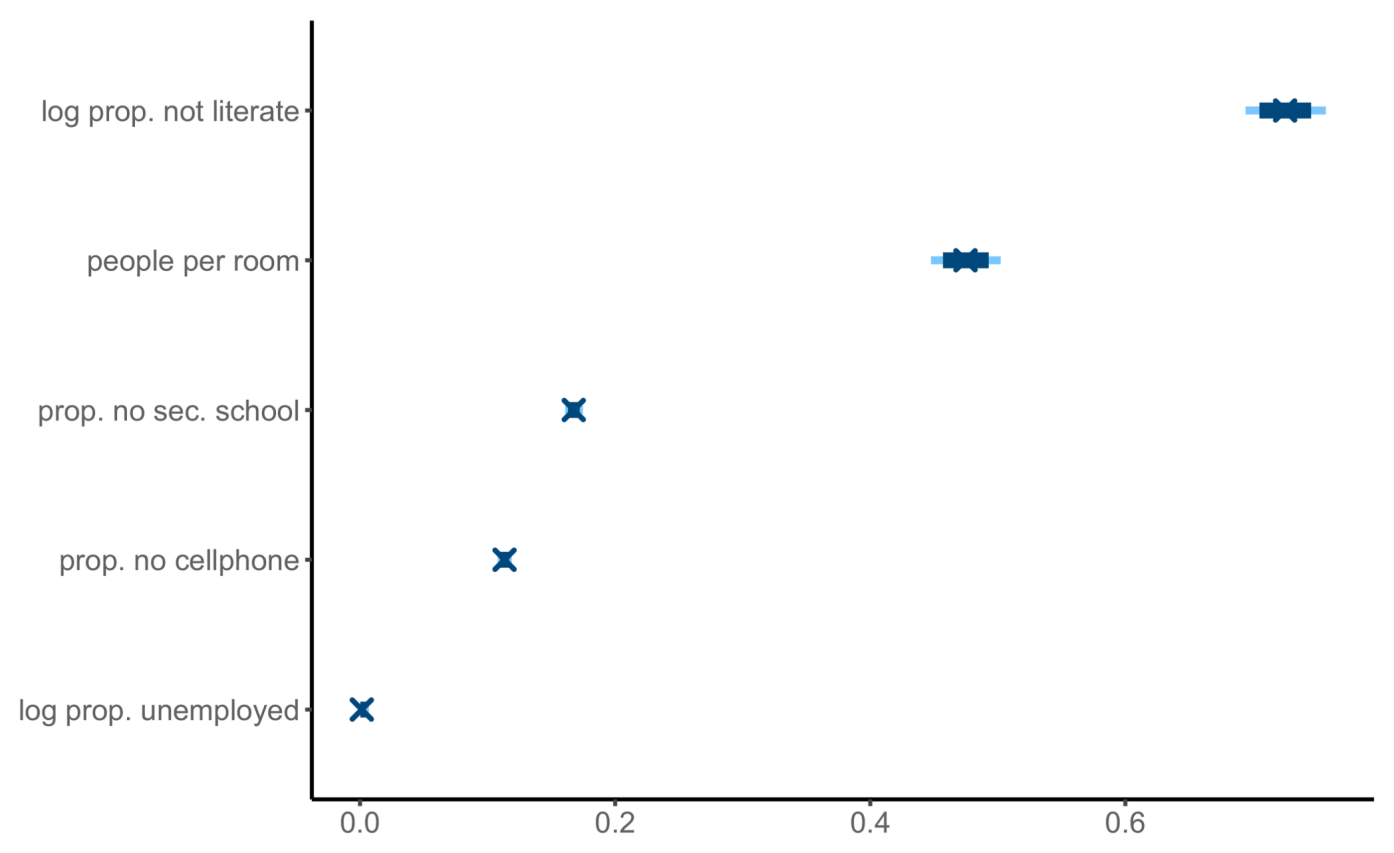
Posterior limits of the 80% (light blue) and 95% (dark blue) credible intervals for the factor loadings (***β***) associated with the continuous variables. A high value indicates the variable better differentiates between high and low SES areas.

Figure 2 suggests that according to the model, the proportion of illiteracy within a household is the continuous variable that best differentiates between low and high SES areas, followed by the ratio of people per room. The proportion of unemployment within the households does not reflect high or low SES areas. According to the census report, GAMA has the highest unemployment rate (8.3%) of all regions of Ghana. However, the distribution of the proportions of unemployment between areas is fairly uniform, explaining the lack of signal detected in the data. Hence, even if this variable is crucial in determining the income of the households and should highly influence their SES, it is not influential in the model as it fails to capture any differences between the areas in the data.

**Figure 3:**
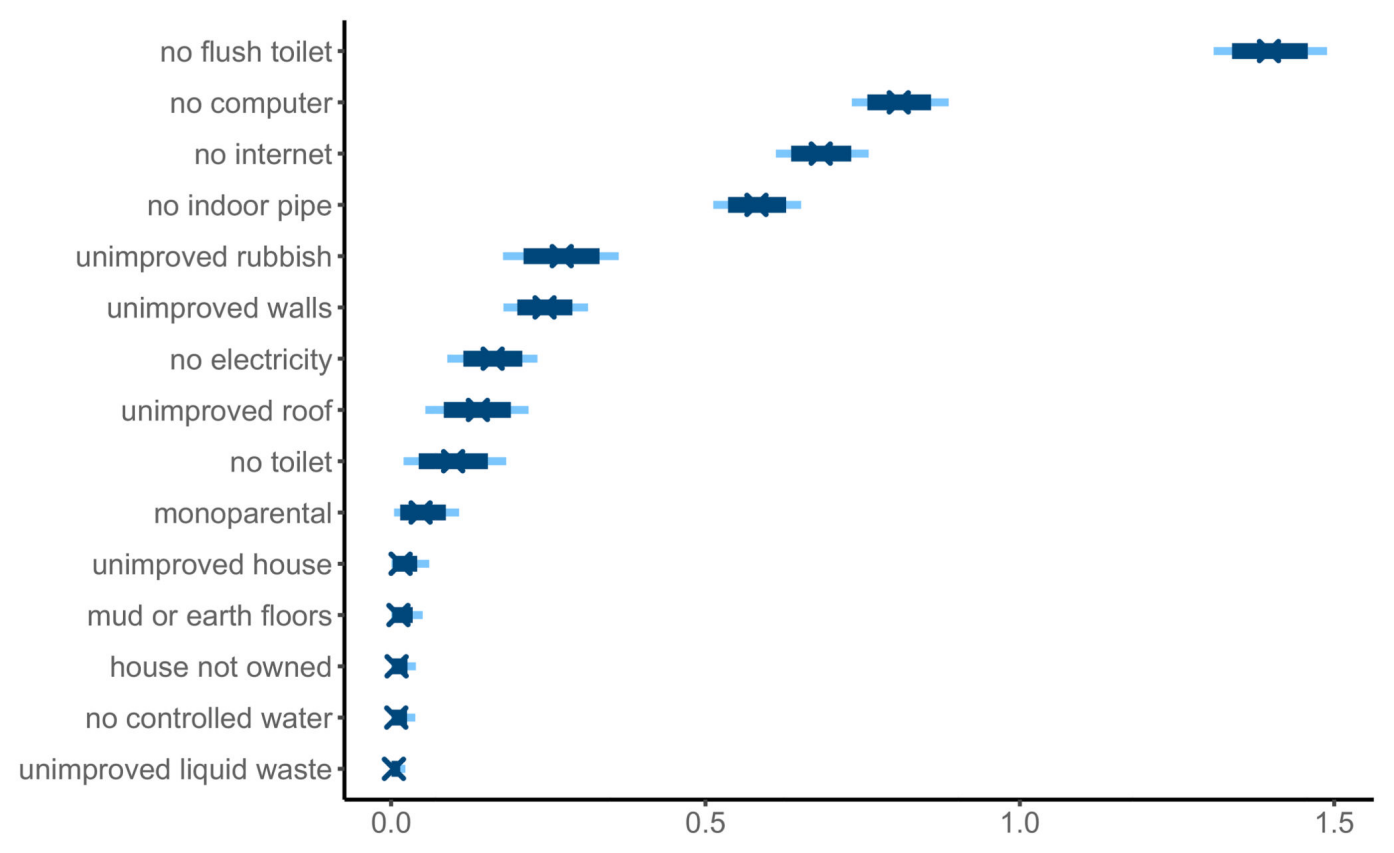
Posterior limits of the 80% (light blue) and 95% (dark blue) credible intervals for the discrimination parameters (***β***) associated with the discrete variables. A high value indicates the variable better differentiates between high and low SES areas.

Figure 3 indicates that not having access to a WC, or flushable toilet, offers the best differentiation amongst dichotomous variables. The presence of an indoor piped drinking water source is also consistently present amongst the highest slope values in all models that were fit. Unimproved rubbish disposal affects sanitation along with the quality of the livelihood and is part of the five most discriminating variables. Not owning a computer and not having access to the internet are withal key determinants of the SES index. These variables can express asset ownership. As internet access encompasses cell phones and internet cafés, it could additionally reflect services provided in the neighbourhood. The least discriminating variables include not owning your house and monoparental households, which are often included in extensive field studies. This suggests that those realities impact a heterogeneous share of the GAMA population, identified as having low SES or not by the model. Proper liquid waste disposal presents a very low slope value and could indicate such services may be the exception rather than the rule. Finally, the credible intervals for dichotomous variables are larger than those presented by continuous variables due to their discrete nature and low counts in some areas.

**Figure 4:**
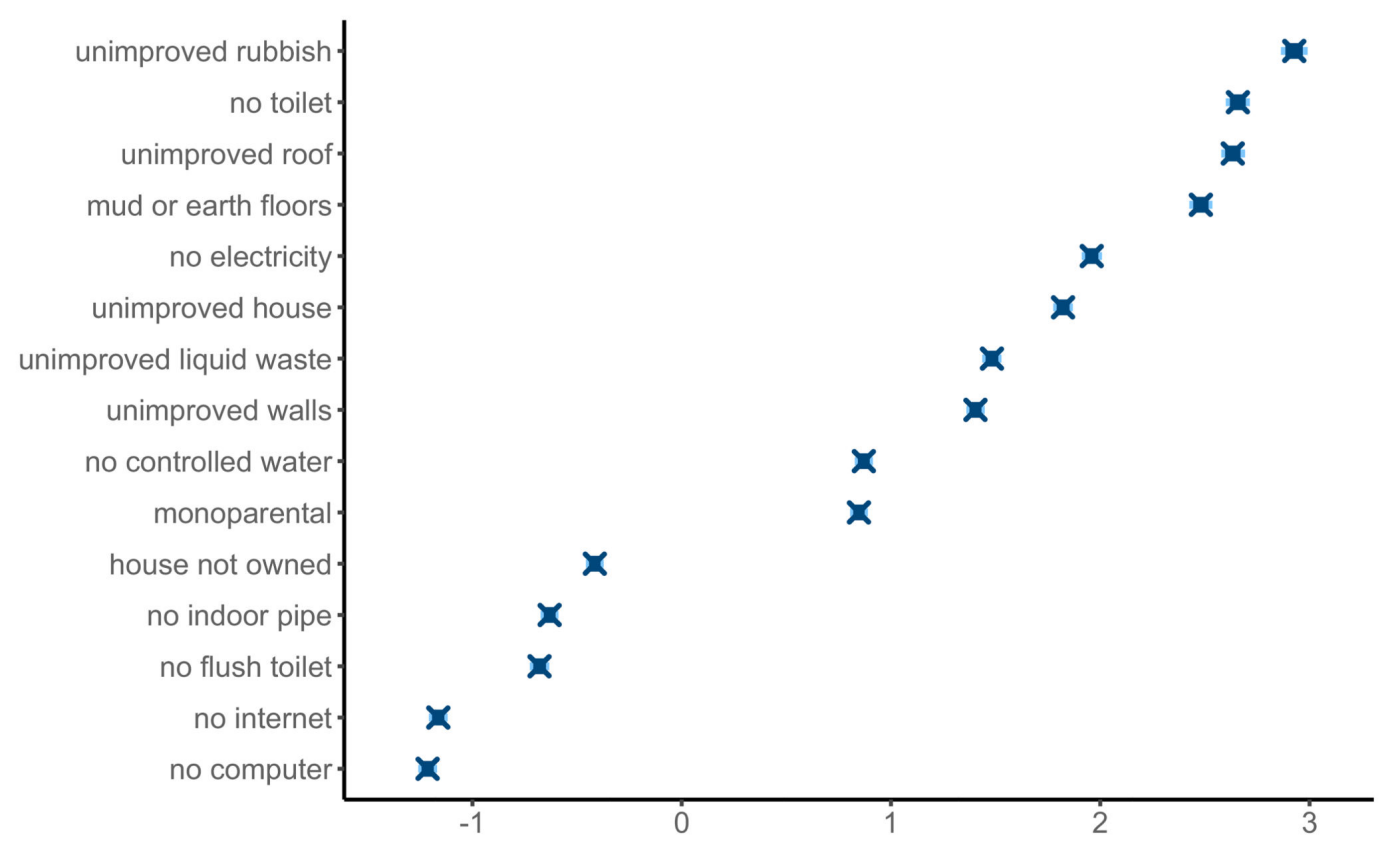
Posterior limits of the 80% (light blue) and 95% (dark blue) credible intervals for overall difficulty parameters (−α^*^) associated with the discrete variables. A high value indicates it is harder to score one for the variable in the areas.

Figure 4 presents the values of the overall difficulties (−α*) of each dichotomous variable. In all models fit, no computer, no internet access, no WC, unimproved water features and no ownership of the house had the lowest difficulty values (or highest intercept value). Whereby, the score of one of these variables reflected a broad range of underlying SES values. Unimproved rubbish disposal, unimproved roof materials and disposing of no toilet facilities had the highest difficulty values. This indicates that a value of one in these variables designates mostly very low SES areas. As they also discriminate well, they are key identifiers of very low SES areas.

**Figure 5:**
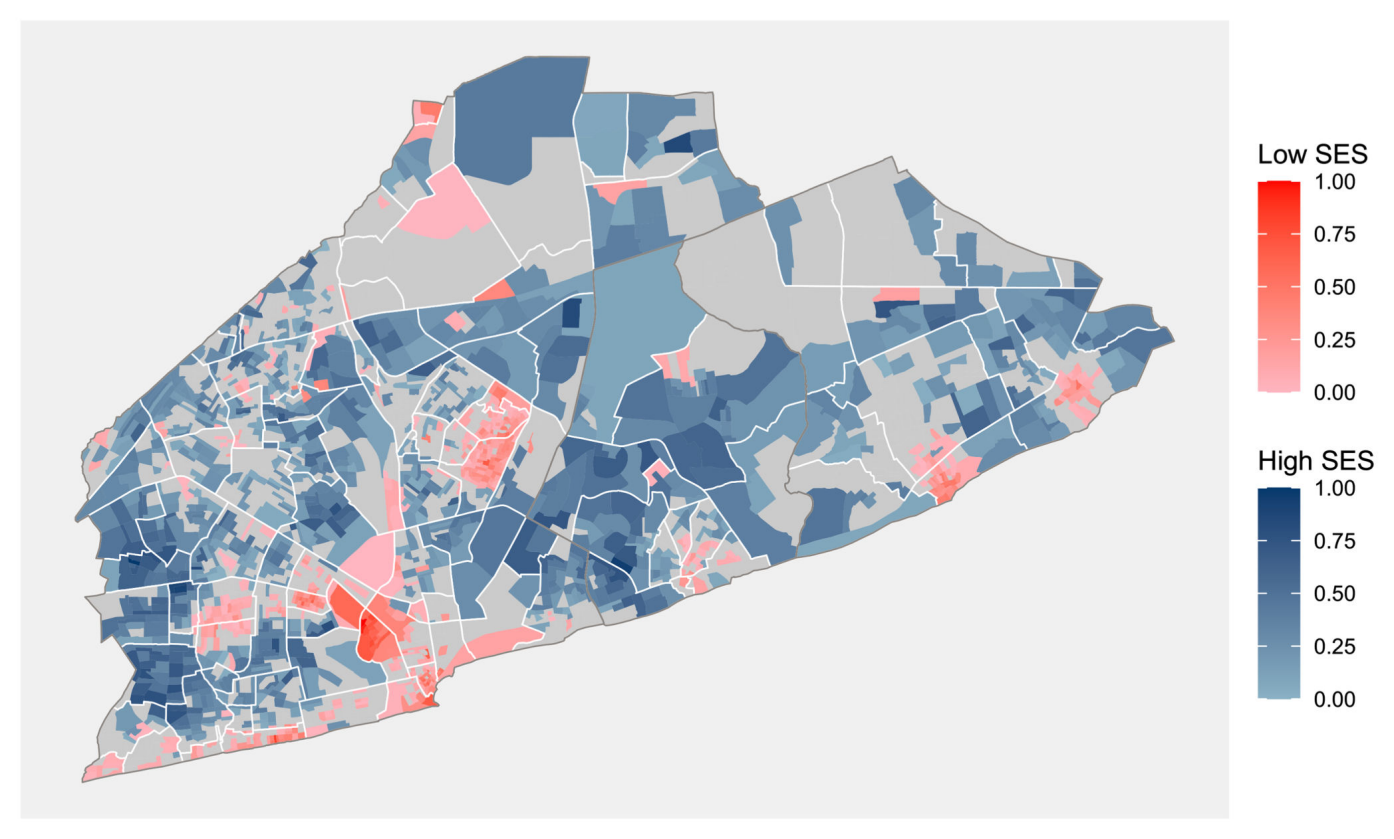
Distribution of the posterior limits of the 90% credible intervals for *θ*_*i*_ across the studied region. Shaded blue areas indicate high socio-economic status (SES), whereas shaded red areas indicate low SES. Grey areas are those that the posterior credible interval included zero, the overall median for ***θ***, that could not be categorized as low or high SES. To ease interpretation, the estimates of each category were re-scaled to take values between 0 and 1. Their true values lie approximately between −2 and 0 for the high SES areas and 0 to 3 for the low SES areas.

Figure 5 identified the most known deprived areas having a low SES, including Jamestown, Nima, Mamobi, and Teshie. Most of the low SES areas are located close to shore. Furthermore, the model also identifies known high SES areas such as Tesano, East Legon, and Roman Ridge. The spatial intensity parameter (*ι*_*θ*_) of the latent index (***θ***) for the proper CAR distribution was estimated at 0.998 (sd = 0.001), showing a strong spatial relationship between neighbouring areas and displaying spatially smooth estimates in the figure. The spatial intensity is estimated very close to 1, suggesting that an intrinsic conditional autoregressive (ICAR) prior can be considered for the indices ***θ***. Section E.3 of online supplementary material shows results based on this prior specification. Further discussion about an ICAR prior for ***θ*** is carried out in Section 4.

**Figure 6:**
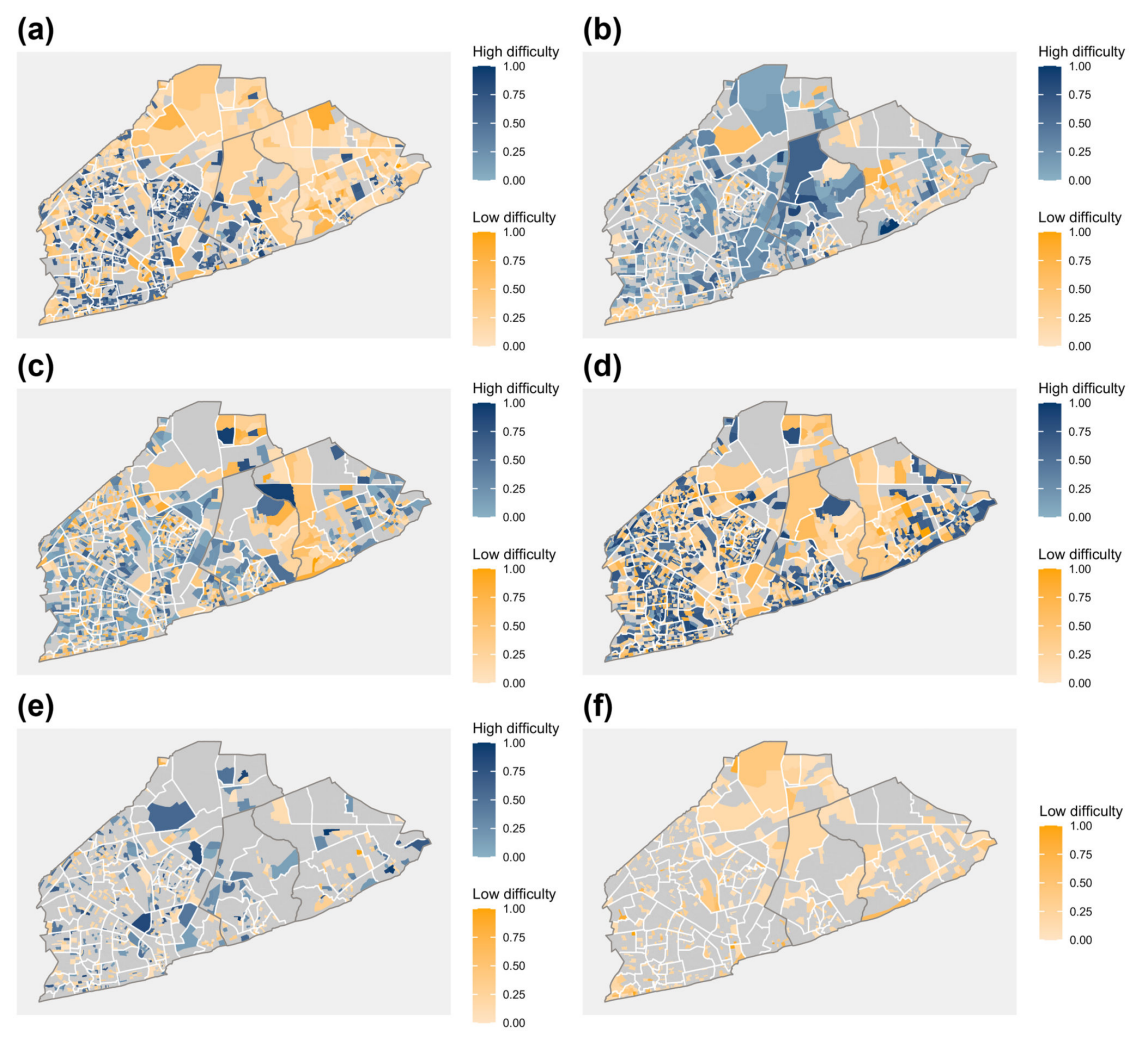
The maps indicate if the posterior limits of the 80% credible intervals for *α*_*ik*_ contained the value of $\alpha_{k}^{*}$ for a given dichotomous variable *k*. The credible intervals for the areas in grey contained the overall difficulty value, while the blue (yellow) areas were strictly greater (lower) than the overall difficulty. The estimates were then re-scaled to take values between 0 and 1. Panels (a) illustrates the variations of unimproved liquid waste management, (b) flushable toilets, (c) indoor piping, (d) unimproved water sources, (e) monoparental households and (f) unimproved rubbish disposal.

Figure 6 illustrates how the values of each of the difficulties in the areas (*α*_*ik*_) differ from the overall difficulty parameter $\left(\alpha_{k}^{*}\right)$. We present 80% credible intervals to allow the spatial pattern of certain variables to be appreciated as some of them, e.g. monoparental households, almost exclusively contained their overall difficulty α* when using 90% intervals. Variables related to infrastructures differed most between areas. Indoor water piping, unimproved liquid waste management and flushable toilets presented the most heterogeneous values. Still, unimproved rubbish disposal presented similar patterns across areas. On the other hand, construction materials did not show particular spatial patterns or high heterogeneity. Finally, monoparental households, access to computers and access to the internet only differed significantly from the overall mean in a few areas, suggesting a hierarchical intercept might not be needed for these variables.

## 4 Discussion

This paper proposed a hierarchical model for mixed continuous and dichotomous variables to estimate area socio-economic indices based on census observations at the household level. We believe our proposed approach improves common practices by accounting for the possible heterogeneity within and between areas and directly accounting for the variation in the number of households per area. According to WAIC, the version of the model best describing the 2010 Ghana census data in GAMA was the hierarchical intercept model with a spatial prior on the latent index. This suggests that the effects of each variable vary across the areas, but that the SES indices of neighbouring areas show a strong spatial correlation, as reflected in the high estimated value of the spatial smoothing parameter. Most of the variables showing high discrepancies in the area-specific intercept values were related to infrastructures, as seen in Figure 6. These differences showed across GAMA but seemed to be clustered. Thus, neighbouring areas probably display similar features like piping and waste management. The areas identified as low SES by the model concur with the findings of Weeks et al. (2012) which used a PCA-based composite index on aggregated data, Engstrom et al. (2019) used satellite imagery and a random forest at the household level averaged at the area level as well as a 2011 UN-Habitat descriptive field study (Assembly & Habitat, 2011). None of these studies considered a model-based approach or the varying number of households per area. Uncertainty around their assessments was not provided either. Most authors also choose to aggregate information, rather than use the data at the household level, ignoring that different areas have a different number of households (Ran et al., 2020). Our model naturally accounts for this and provides measurements of uncertainty about the resultant estimates.

**Figure 7:**
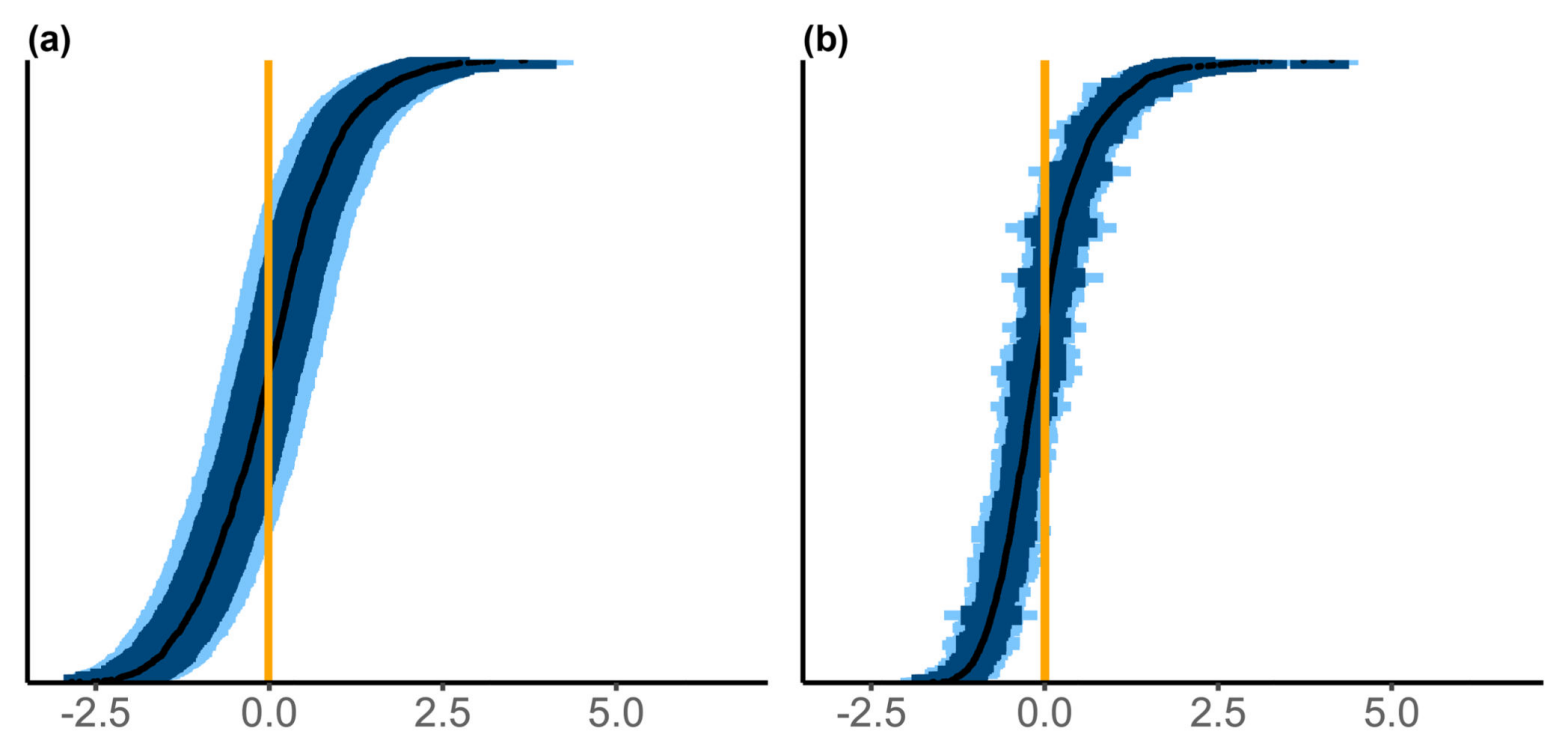
Comparison of the 95% posterior credible intervals for the latent indices when using factor analysis based on observed proportions of the variables across EA’s (a) and the proposed hierarchical model, that uses information at the household level within each EA (b).

Figure 7 compares the distributions of the latent SES indices and their uncertainty when using traditional factor analysis on aggregated data or the hierarchical model proposed. The varying width of credible values is directly linked to the number of households in each area, ignored in the summaries obtained under the model in the left-hand side. Another advantage of the proposed model lies in the estimation of different difficulty parameters per area. This not only provides information on how the different variables affect each area, but it also allows us to borrow strength across the map to estimate the overall parameters.

Out of the 20 observed variables, the number of people per room, illiteracy, access to water piping and flushable toilets differentiated high and low SES areas the best, three of which are part of the five criteria the UN-Habitat uses to describe unimproved housing (UN-Habitat, 2004). Figure 3 indicates that not having access to a WC, or flushable toilet, offers the best differentiation amongst dichotomous variables. This variable not only reflects households connected to the piped network but also those having access to improved sanitation, reducing health hazards exposure (Bartram & Cairncross, 2010; Organization, 2016). The urban poor are also more likely to require the use of public toilets, increasing risks of diseases (Boadi, 2004). The presence of an indoor piped drinking water source is also consistently present amongst the highest slope values, which is a key determinant in the quality of livelihood and health (Bain et al., 2014; Bartram & Cairncross, 2010; Moore et al., 2003; Organization, 2016). Access to quality water features is limited in many neighbourhoods in GAMA. The lack of infrastructures and reliability of water sources might explain this deficiency (Awuah et al., 2009; Stoler et al., 2015). Furthermore, being connected to the piped water network and flush toilet is mostly restricted to high-income neighbourhoods (Boadi, 2004). Variables best-discerning statuses also included unimproved rubbish disposal, which affects sanitation along with the quality of the livelihood. Poor waste management is likewise associated with health hazards (Lamond et al., 2012; Louw et al., 2019). Rubbish disposal in low-income neighbourhoods is usually carried by individuals charging a fee (Boadi, 2004). Most households in these areas may not be able to afford such services. Liquid waste disposal did not seem to be common practice. Songsore (2008) and Awuah et al. (2009) pointed out in their Accra case studies that a small portion of households practiced safe liquid waste disposal, and a vast majority of them qualified as wealthy. Access to technologies played a key role in the model as well. Not only do they allow users to get information but also access to the Internet has also been linked to mobility; either through emigration opportunities or SES mobility (Burrell, 2009). On the contrary, ownership of tenure did not provide much information regarding area SES, despite being identified as a key factor by the UN (UN-Habitat, 2004). The housing crisis in Accra has been studied by authors such as Owusu-Ansah et al. (2018) and Yankson (2012), proposing lack of tenure and high rent cost explain this widespread rental pattern. The housing crisis in Ghana has affected mostly mid to low-income households due to the lack of available, affordable and adequate housing (Gillespie, 2018).

It is common practice to assess SES using uniform or predefined weights for each variable. These weights sometimes take into account the data of the studied region but rarely vary within it, see for example Filmer and Pritchett (2001); Flanagan et al. (2011). Our results suggest such practices could be misleading, preferring hierarchical modelling. Furthermore, our model enabled the use of both dichotomous and continuous variables, keeping as much information as they could offer. Yet, some data still had to be transformed into dichotomous entries due to low-count categories. This dichotomization was done according to common practices but were subjective choices. Moreover, the use of the median to separate between low and high SES indices was an arbitrary choice that could be improved by consulting with other specialists in order to better capture mid-income areas.

As pointed out by one of the reviewers, the correlation parameter, *ι*_*θ*_, of the proper CAR prior for the factor (see Section 2.1) is estimated very close to **1**, suggesting that an ICAR prior could be considered for *θ*_*i*_. The ICAR is an improper prior distribution, and a sum-to-zero constraint can be imposed to guarantee a proper posterior distribution. The model with an ICAR prior was fitted in Stan and Nimble (de Valpine et al., 2017). As previously mentioned, Stan uses Hamiltonian Monte Carlo methods to obtain samples from the resultant posterior distribution. Nimble, on the other hand, uses a Gibbs sampler with some steps of the Metropolis–Hastings algorithm. As the chains were taking long to converge in Stan, we wanted to double-check if another MCMC algorithm would provide signs of convergence in less amount of time. Both samplers required a large number of iterations to reach reasonable effective sample sizes and values of $\hat{R}$ (Vehtari et al., 2021) for the factor loadings. Abellan et al. (2007) seem to have encountered similar issues in a model for continuous outcome only. Their factor model with an ICAR prior for the factor ran five times more iterations than other versions of their proposed model. We believe this convergence issue is aggravated when there are outcomes of different types, continuous and discrete. Abellan et al. (2007) do not provide details about the resultant effective sample sizes and other convergence diagnostic measurements. Section D.3.1 online supplementary material shows results from a simulation study assuming an ICAR prior for ***θ***. Results suggest that we are able to recover the values of the parameters used to generate the data, but the chains take long to converge. Section E.3 of online supplementary material shows the results for the Ghana data analysis assuming an ICAR prior for ***θ***. The MCMC under the ICAR prior took much longer to converge when compared to the proper CAR prior; the resultant effective sample sizes were relatively small (smallest ESS was 276) even after long runs of the MCMC. Yet, considering that convergence was reasonably satisfactory, the model with a proper CAR provides the smaller value of the WAIC (see Section E.3 of online supplementary material). As our sample size is quite large (more than 56 K observations in total) running very long chains becomes time-sensitive (it took 44.5 h for two chains to run 20,000 iterations each). For these reasons, we focused on the results under the proper CAR prior. For the class of proposed models in this paper, we recommend that both the proper CAR and ICAR prior specifications for ***θ*** be considered. However, the modeller should consider running the ICAR model for a large number of iterations to obtain acceptable effective sample sizes.

The model could be further improved by including variables at the neighbourhood level, such as a greenery index or population density. Elevation and flood risks would also be a good addition, as most low SES areas identified were close to shore or a river bank (Amoako, 2012). To check the sensitivity of the estimated quantities to the probit link function for the dichotomous variables, we also fitted models that assumed a logit function instead. Although not shown here, the results were very similar to the ones shown in Section 3. Overall, this paper offers a significant improvement to traditional area socio-economic status estimation methods by proposing a model-based approach capable of handling mixed data, correctly accounting for the number of households in the areas and capturing within and between area heterogeneity.

## Supplementary Material

Supplementary materialis available online at *Journal of the Royal Statistical Society: Series A*.

Supplementary Material

## Figures and Tables

**Table 1:** Description of the four fitted models to the Accra dataset

Model	Structure of Y*	Prior for *α*	Prior for *θ*
Hierarchical, spatial (HS)	$y_{ijk}^{*}=\alpha_{ik}+\beta_{k}\theta_{i}+\epsilon_{ijk}$	$\begin{array}{c} \alpha_{ik}\sim N\left(\alpha_{k}^{*},1\right)\\ \alpha^{*}\sim N(0,I) \end{array}$	$\theta \sim N(0,{\left(D-{ı}_{\theta }W\right)}^{-1})$
Hierarchical, non-spatial (H)	$y_{ijk}^{*}=\alpha_{ik}+\beta_{k}\theta_{i}+\epsilon_{ijk}$	$\begin{array}{c} \alpha_{ik}\sim N\left(\alpha_{k}^{*},1\right)\\ \alpha^{*}\sim N(0,I) \end{array}$	***θ*** ~ *N*(0, *I*)
Non-hierarchical,spatial (S)	$y_{ijk}^{*}=\alpha_{k}+\beta_{k}\theta_{i}+\epsilon_{ijk}$	***α*** ~ *N*(0, *I*)	$\theta \sim N\left(0,{\left(D-{ı}_{\theta }W\right)}^{-1}\right)$
Simplest (M0)	$y_{ijk}^{*}=\alpha_{k}+\beta_{k}\theta_{i}+\epsilon_{ijk}$	***α*** ~ *N*(0, *I*)	***θ*** ~ *N*(0, *I*)

*Note*. Note that ***D***_***N***×***N***_ is a diagonal matrix with elements set to the total number of neighbours each EA has and ***W***_***N***×***N***_ denotes a 0–1 adjacency matrix.

**Table 2:** Values of WAIC and its components, the effective number of parameters (*p*_*WAIC*_) and the log-pointwise predictive density (*lppd*), for each of the fitted models

Model	*p*_*waic*_	*lppd*	WAIC
HS	20,457	562,109	*1,165,130*
H	20,837	561,774	1,165,220
S	4,072	630,412	1,252,680
M0	4,349	630,247	1,251,796

*Note*. The value in italics points out the best model among the fitted ones.

## Data Availability

For data access please write to alexandra.schmidt@mcgill.ca.
