# Supplementary material for: Mapping socio-economic status using mixed data: a hierarchical Bayesian approach
Source: J R Stat Soc Ser A Stat Soc. Author manuscript; Available in PMC 2025 Feb 27. (PMC7617442; doi:10.1093/jrsssa/qnae080)
Supplement: Supplementary Material [file EMS201701-supplement-Supplementary_Material.pdf]

Supplementary Material of "Mapping socio-economic status using mixed data: a hierarchical Bayesian approach"

Gabrielle Virgili-Gervais[1], Alexandra M. Schmidt*[1], Honor Bixby[2], Alicia Cavanaugh[3], George Owusu[4], Samuel Agyei-Mensah[5], Brian Robinson[3], and Jill Baumgartner[1]

[1]Department of Epidemiology, Biostatistics and Occupational Health, McGill University

[2]Institute of Public Health and Wellbeing, University of Essex

[3]Department of Geography, McGill University

[4]Institute of Statistical, Social & Economic Research, University of Ghana

[5]Department of Geography and Resource Development, University of Ghana

August 1, 2024

This document provides further details about the proposed model and results described in the main text of the paper.

* *Corresponding author*: Alexandra M. Schmidt, Department of Epidemiology, Biostatistics and Occupational Health, McGill University, 2001 McGill College Avenue, Suite 1200, Montreal, QC, Canada, H3A1G1. *E-mail*: alexandra.schmidt@mcgill.ca *Homepage:* http://alex-schmidt.research.mcgill.ca/

A Variables used in the models

Name	Type	Description
**Social aspects**		
Proportion of illiteracy	Continuous	Proportion of the household who could not read or understand the meaning of a given phrase.
Proportion with at most primary school education	Continuous	Proportion of the household for whom the highest level of education achieved was at most primary school. People who never attended school were also included in this category.
Proportion with no cellphone	Continuous	Proportion of the household owning a mobile phone.
No computer	Dichotomous	Indicates if the household owns at least a computer.
No internet	Dichotomous	Indicates if the household has access to internet at home, on any device, or to an internet cafe.
**Livelihood aspects**		
Unimproved home	Dichotomous	Indicates households living in huts, tents or unfinished buildings.
Mud or earth floors	Dichotomous	Indicates that the house's floors are made of non-durable materials such as mud or earth.
Unimproved roof	Dichotomous	Indicates the house's roof is made of other materials than metal, slate or cement.
Unimproved walls	Dichotomous	Indicates the house's walls are made of other materials than brick or cement.
No electricity	Dichotomous	Indicates households without access to electricity.
No indoor pipe	Dichotomous	Indicates households with no indoor water pipe.
No controlled water	Dichotomous	Indicates households without access to a city-controlled water source like a pipe or a protected well.
No toilet	Dichotomous	Indicates households without access to a toilet, shared or not.
No WC	Dichotomous	Indicates households without a flush toilet.
No rubbish disposal	Dichotomous	Indicates households requiring to dump indiscriminately or to bury their rubbish.
Liquid waste	Dichotomous	Indicates households who do not dispose of liquid waste in a sewage or drainage system.
**Economical aspects**		
People per room	Continuous	Ratio of the total number of people in the household divided by the number of rooms in the house.
Mono-parental	Dichotomous	Indicates if the head of the household is a single parent.
Proportion of unemployment	Continuous	Proportion of the household being unemployed at the time of the census.
House not owned	Dichotomous	Indicates the household does not own their house.

B Summaries of the Variables

Variable		Overall ($\sum_{n_i=1}^{N} = 56231$)
**Social aspects**		
Proportion of illiteracy		
	mean (sd)	0.10 (0.25)
	median [min, max]	0.00 [0.00, 1.00]
Proportion with at most primary education		
	mean (sd)	0.20 (0.33)
	median [min, max]	0.00 [0.00, 1.00]
Proportion with no cellphone		
	mean (sd)	0.20 (0.31)
	median [min, max]	0.00 [0.00, 1.00]
No computer		
	0	9 742 (17.3%)
	1	46 489 (82.7%)
No internet		
	0	9 742 (17.3%)
	1	46 489 (82.7%)
**Economical aspects**		
People per room		
	mean (sd)	2.27 (1.65)
	median [min, max]	2.00 [0.04, 24.0]
Mono-parental		
	0	44 094 (78.4%)
	1	12 137 (21.6%)
Proportion of unemployment		
	mean (sd)	0.05 (0.17)
	median [min, max]	0.00 [0.00, 1.00]
House not owned		
	0	20 548 (36.5%)
	1	35 683 (63.5%)

Variable		Overall ($\sum_{n_i=1}^{N} = 56231$)
**Livelihood aspects**		
Unimproved home		
	0	51 400 (91.4%)
	1	4 831 (8.6%)
Mud or earth floors		
	0	53 863 (95.8%)
	1	2 368 (4.2%)
Unimproved roof		
	0	54 796 (97.4%)
	1	1 435 (2.6%)
Unimproved walls		
	0	48 172 (85.7%)
	1	8 059 (14.3%)
No electricity		
	0	52 705 (93.7%)
	1	3 526 (6.3%)
No indoor pipe		
	0	17 548 (31.2%)
	1	38 683 (68.8%)
No controlled water		
	0	39 031 (69.4%)
	1	17 200 (30.6%)
No toilet		
	0	54 513 (96.9%)
	1	1 718 (3.1%)
No WC		
	0	18 205 (32.4%)
	1	38 026 (67.6%)
No rubbish disposal		
	0	55 013 (97.8%)
	1	1 218 (2.2%)
Liquid waste		
	0	46 969 (83.5%)
	1	9 262 (16.5%)

C Correlation of the aggregated variables

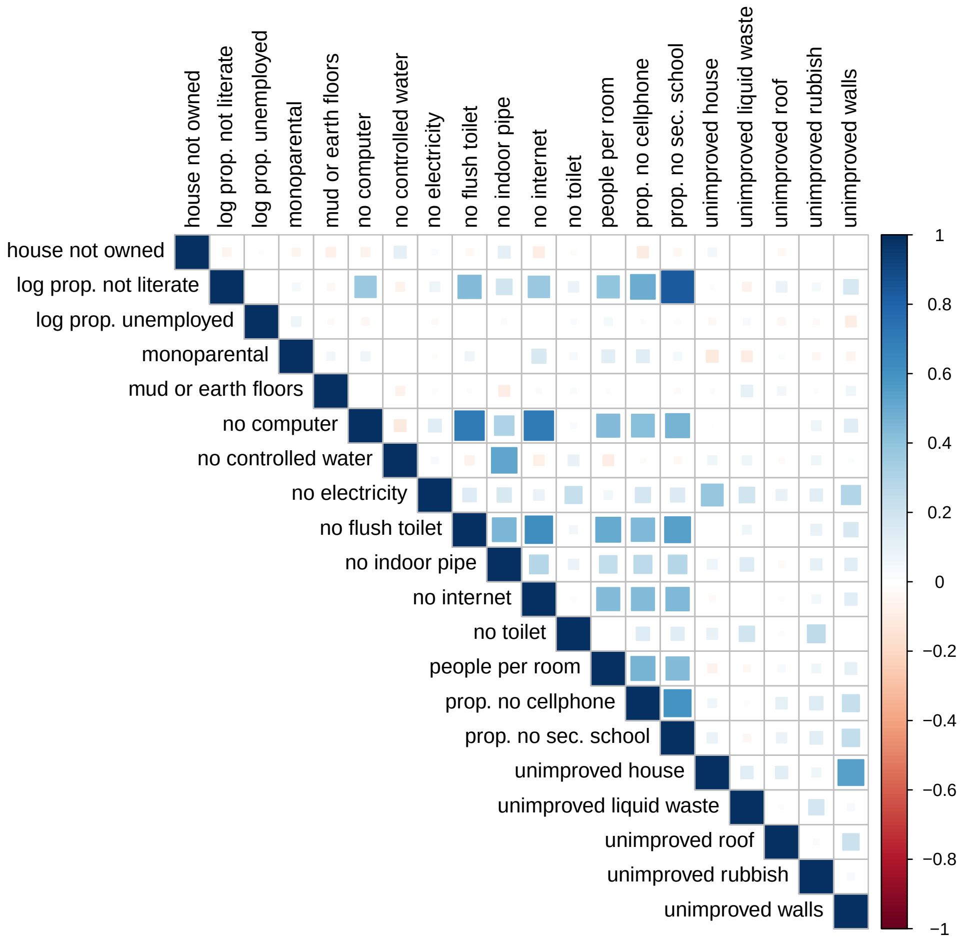

The correlation shown is the one observed when pooling the individual-level variables at the neighbourhood-level in the Ghana dataset. The average of the continuous variables and the proportion across households were considered. Most of the variables are correlated, which is suitable for fitting a factor analysis model. Almost all the variables show a positive correlation, or a very small negative one. In factor analysis and item response theory, these positive correlations at the area-level would be translated into a higher value of the latent trait measured conjointly by these variables.

D Simulation studies

To better understand parameter recovery, identifiability issues and the impact of prior specifications, we conducted several sets of simulation studies. For each scenario considered, we generated 100 datasets. Each dataset was composed of a subset of 200 adjacent neighbourhoods from the Ghana data, which comprised 5,195 observations. All neighbourhoods shared borders with at least another one. For each observation, we generated 15 dichotomous and 5 continuous variables.

The true spatial intensity parameter was set to 0.9. In the true underlying model, $\boldsymbol{\theta}$ followed a proper CAR, with a hierarchical structure on the difficulty parameters and either separate difficulty variance parameters or variance set to one. The true parameter values used to generate the data were the estimates obtained from fitting the model to the full Ghana data when allowing free variance parameters for the difficulty. Because we wanted to test Schliep and Hoeting (2013) identification restrictions for the difficulty, we fixed the variance of the difficulty of the first dichotomous variable to one. The true underlying SES was generated from a proper CAR distribution with a precision parameter equal to one. Each continuous variable was simulated from a normal distribution as in equation (2) while dichotomous variables were simulated using a probit model. For each scenario and each simulated dataset, we ran the respective `Stan` model for 10,000 iterations of one MCMC chain and saved the full samples. Each time, values of α_{ik} and the observed variables were generated.

D.1 Estimating the spatial precision

Here we go back to the point discussed in the paragraph about Identifiability Issues in Section 2.2 of the main text. Wang and Wall (2003), Schliep and Hoeting (2013) and Higgs and Hoeting (2010) have all pointed out the need to set the precision parameter to one, both in the case of areal data (with a CAR prior for the factor) and point-referenced data

(with a Gaussian process prior for the factor). We ran a simulation study wherein this parameter was estimated with a Cauchy(0,1) prior for the variance. The true value used to generate the data was one. In the model fit to the datasets, the variance of the prior distribution of the hierarchical difficulty parameters was set to one for all variables, that is $\alpha_{ik} \sim \text{Normal}(\alpha_k, 1)$, for $k = 1, \cdots, p$, and $i = 1, \cdots, N$. Despite being able to recover the spatial intensity parameter, the spatial precision was overestimated, with a median of medians around 3.3 which ranged from 2.04 to 4.95. The factor loadings (β_i) were also overestimated, though their relative ordering was correct.This confirms the results shown in the paragraph about identifiability issues in Section 2.2. In the case of dichotomous variables, as De Oliveira (2000) discussed in his paper, only one parameter should be estimated regarding the correlation structure of a latent variable as the other would be near-non-identifiable. not be possible to identify it when a spatial structure is assumed. The simulations we ran seem to confirm his point.

D.2 Estimating the variance of the difficulty parameters ($\boldsymbol{\sigma}_\alpha$)

Another simulation study we performed was to check if we were able to estimate the standard deviation of the hierarchical prior for α_{ik}. When trying to estimate a CAR structure on $\boldsymbol{\theta}$ and a hierarchical structure on $\boldsymbol{\alpha}$ with free, half-Cauchy(0,1) priors for the standard deviation parameters, it seems we are not able to recover all of them. Their estimates seem to be pushed towards one. Unfortunately, fixing one of their values to one as Schliep and Hoeting (2013) did not improve the fit.

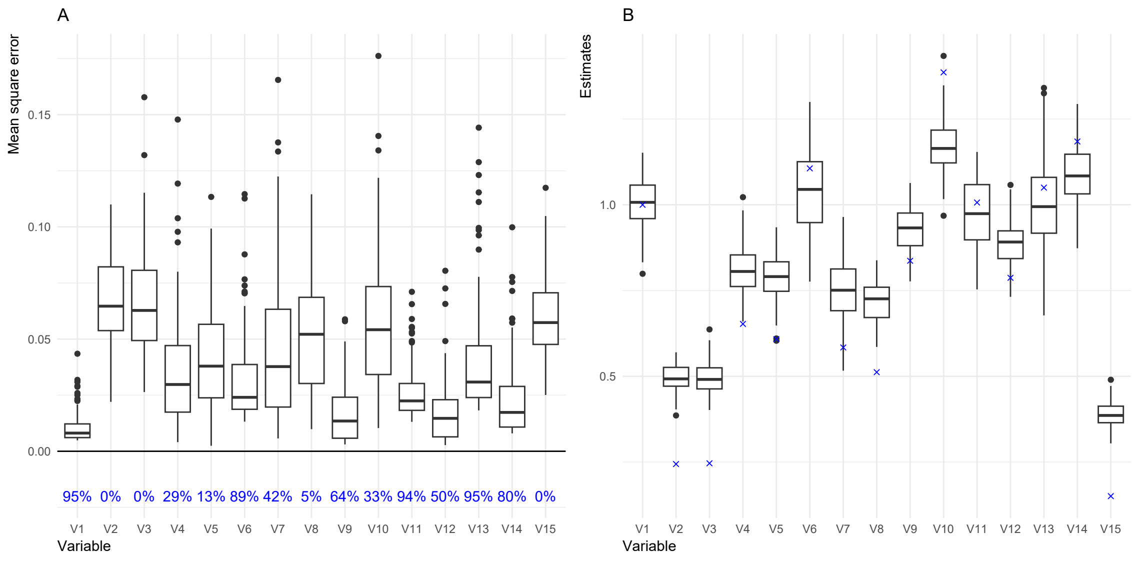

Figure 1: Simulation study results for $\boldsymbol{\sigma}_\alpha$ when $\boldsymbol{\theta}$ follows a proper CAR distribution. In panel A, percentages in blue indicate the number of times the true parameter was part of the posterior 95% credible intervals while the boxplots show the mean square errors. True values are shown as blue crosses in panel B.

When the true value was close to one, we could see coverage of the credible intervals close to what would be expected, for instance, variables 1, 6, 11 and 13 show acceptable recovery in Panel **??**. The coverage seems to decline the further the true value is from one. Overall, the mean square errors across the datasets hover around 0.05.

We further investigated this phenomenon when $\boldsymbol{\theta}$ follows a standard normal distribution. Similar results were observed. It seems we are not able to disentangle the two sources of random variation, coming from the observations and the random difficulty. In our simulation study, each area contains between 5 and 99 observations, with a median of 22. In the Ghana dataset, the number of households in areas varies from 1 to 269 with a median of 22. Because about 25% of the considered areas contain less than 15 observations, we may lack information to identify so many parameters.

In all, when generating the data using varying true values for $\boldsymbol{\sigma_\alpha}$ and fitting a model with $\boldsymbol{\sigma_\alpha}$ kept free, the only parameter we are not able to recover is $\boldsymbol{\sigma_\alpha}$ itself. When generating data with varying true values of $\boldsymbol{\sigma_\alpha}$ and fitting a model with $\boldsymbol{\sigma_\alpha} = \mathbf{1}$, we can recover all

parameters. As such, we chose to keep it fixed at one to estimate the model on the Ghana data.

D.3 Results from the spatial hierarchical model

A third simulation study was performed to investigate the parameter recovery of the best-fitting model on the Ghana dataset, one where the difficulty parameters ($\boldsymbol{\alpha}$) are hierarchically distributed with a variance fixed at one and the latent index ($\boldsymbol{\theta}$) follows a proper CAR prior. Overall, we can recover all parameters of interest in the model. Credible intervals offer coverage rates close to the expected ones (around 95 %) and posterior medians are shown to adequately estimate the parameters. Variables with low discrimination parameters might be over-estimated and should be interpreted with caution.

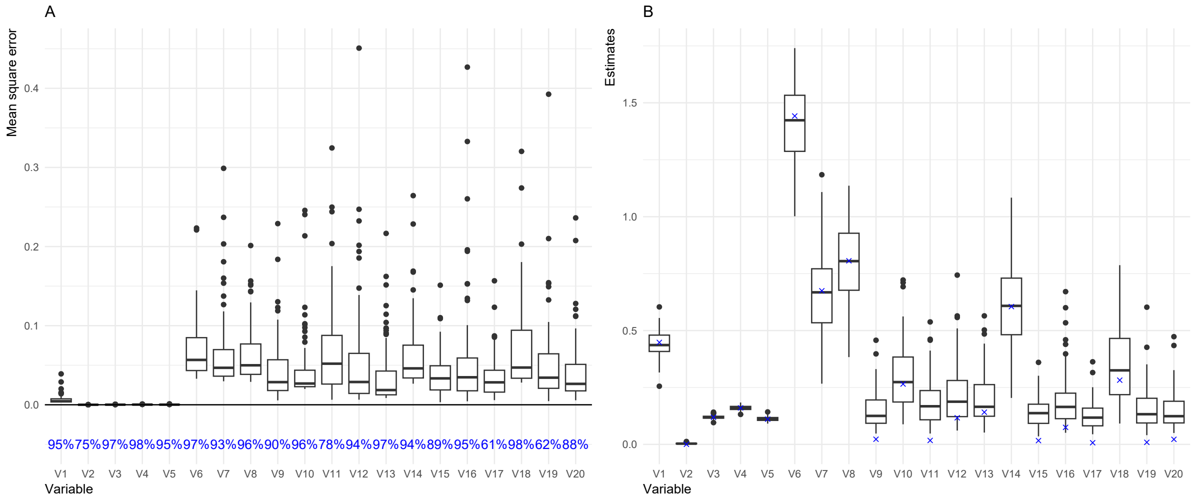

Figure 2: Simulation study results for $\boldsymbol{\beta}$ when $\boldsymbol{\theta}$ follows a proper CAR distribution. In panel A, percentages in blue indicate the number of times the true parameter was part of the posterior 95% credible intervals. True values are shown as blue crosses in panel B. The five first variables correspond to continuous variables while the rest refer to dichotomous ones

Factor loadings of the continuous variables are well estimated and do not show much variation across generated datasets. Discrimination parameters of dichotomous variables tend to have wider intervals of credible values. Variables with high discriminative abilities are recovered. When looking at variables 6, 7, 8, and 14, we notice their ranking compara-

tively to the other variables is recovered, but their medians cover a wider interval of values across datasets. The coverage rate of their credible intervals is however adequate. When the discrimination of a variable is very close to zero, it tends to be slightly overestimated. Variables 11, 17, and 19 in particular show inadequate coverage rates of their posterior credible intervals going as low as 61%. Because they have quasi-null discrimination, the slope of the item characteristic curve is almost null, exhibiting a straight horizontal line. Hence, these variables are not of interest in our case study, as they do not differentiate well between areas. The results from our simulation study show we should interpret low-discrimination variables with caution, as they may have been over-estimated.

The variance parameters of the continuous variables are well recovered and show high posterior precision. Their coverage rates are at least 94% and MSE of less than 0.001. The spatial intensity parameter is also well recovered, though slightly underestimated. Its coverage rate is 97% with MSE around 0.015. The median of its posterior medians over 100 datasets sits around 0.85 (its true value was 0.9).

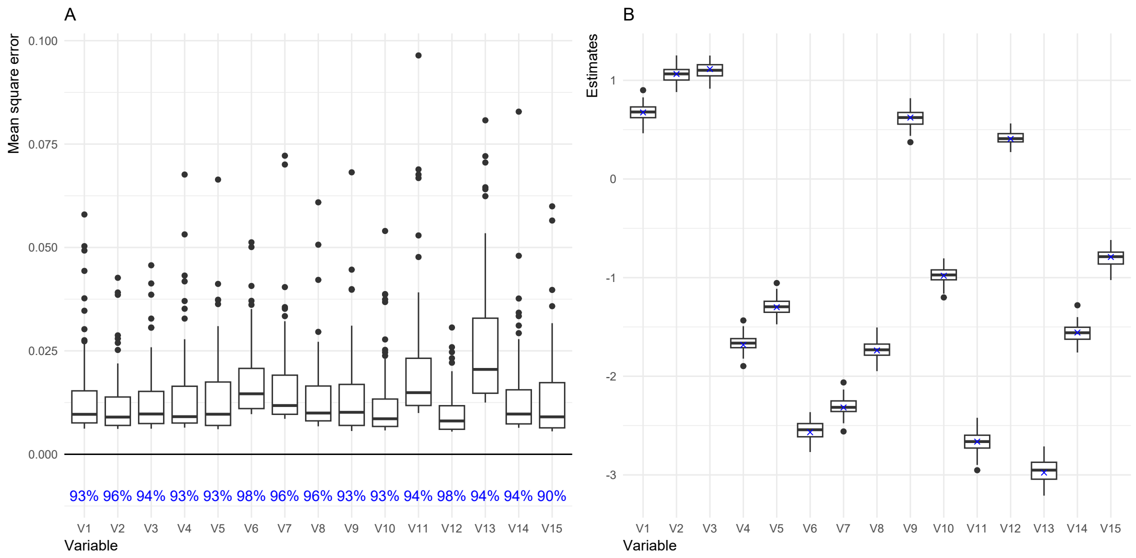

Figure 3: Simulation study results for $\boldsymbol{\alpha}^*$ (the overall mean of the hierarchical difficulties) when $\boldsymbol{\theta}$ follows a proper CAR distribution and the variance of the hierarchical difficulties equals one. Percentages in blue in panel A indicate the number of times the true parameter was part of the posterior 95% credible intervals. True values are shown as blue crosses in panel B.

The overall average difficulty parameters are well recovered. When the data is simulated with free variance and a fixed variance of one is imposed in the model, they exhibit decent behaviours. Most coverage rates hover between 90% to 95% except for dichotomous variables 7 and 8 which are around 60%. Their MSE varies from 0.01 to 0.04 and their medians tend to be slightly off target, without any particular pattern.

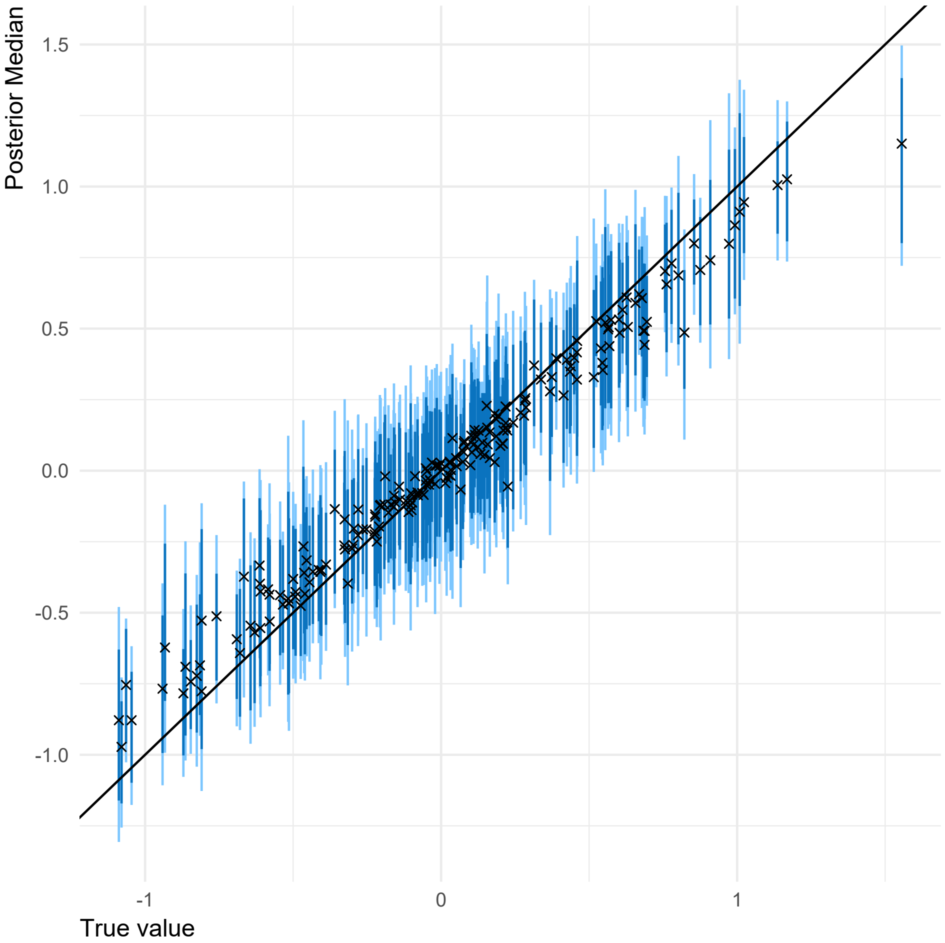

Figure 4: Simulation study results when $\boldsymbol{\theta}$ follows a proper CAR distribution and the variance of the hierarchical difficulties equals one. Light blue segments represent 95% intervals for the median values, while dark blue segments represent 90% intervals. True values are shown as black crosses.

Figure 4 shows the scatter plot of the true value of the index, θ_i versus the summary of the estimated values under each of the 100 datasets. Even if they present a slight attenuation from their true spread, posterior median intervals almost always contain the true value.

Medians of posterior medians show a correlation with true values of 0.9. Overall, we can recover the parameters in the model in a satisfactory way.

D.3.1 Imposing an ICAR prior to the factor θ_i

Finally, we ran a simulation study similar to the one described in Section D.3 but now the latent factor $\boldsymbol{\theta}$ was simulated from an intrinsic conditional auto-regressive (ICAR) distribution. We followed the conditional by kriging algorithm described in Rue and Held (2005) to generate the latent factor.

Then we fitted the model assuming the ICAR prior for $\boldsymbol{\theta}$ imposing a soft sum-to-zero constraint to guarantee that the resultant posterior is proper. The goal was to investigate if we were able to estimate the parameters in the model. The results were similar to the ones obtained assuming a proper CAR for θ. See panels of Figures **??** and 6.

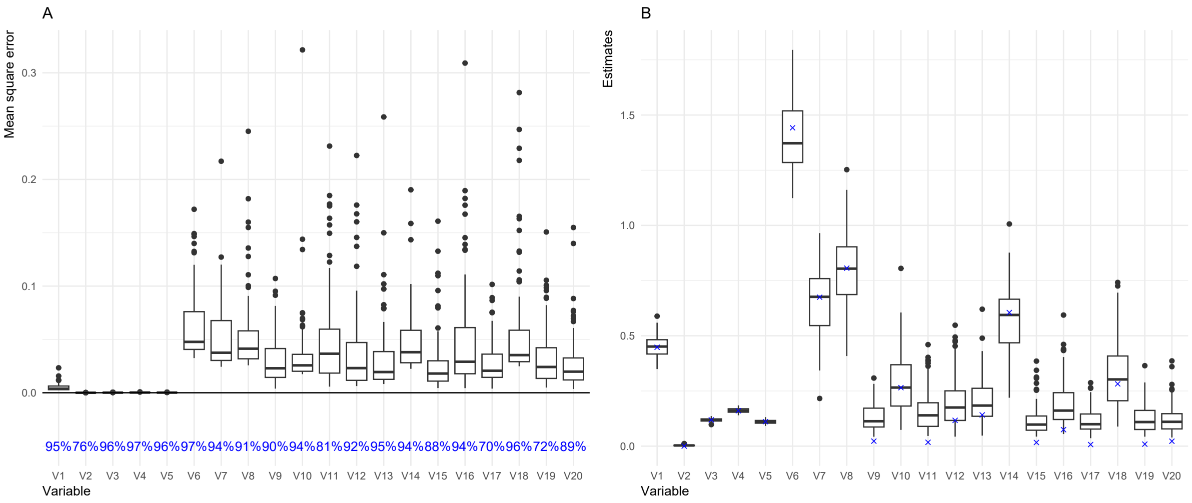

Figure 5: Simulation study results for $\boldsymbol{\beta}$ when $\boldsymbol{\theta}$ follows an ICAR distribution. Percentages in blue indicate the number of times the true parameter was part of the posterior 95% credible intervals. True values are shown as blue crosses in b). The five first variables correspond to continuous variables while the rest refer to dichotomous ones.

The discrimination parameters display a slightly better coverage rate than the proper CAR prior and at times smaller MSEs. The results for the difficulty parameters and standard deviations are alike.

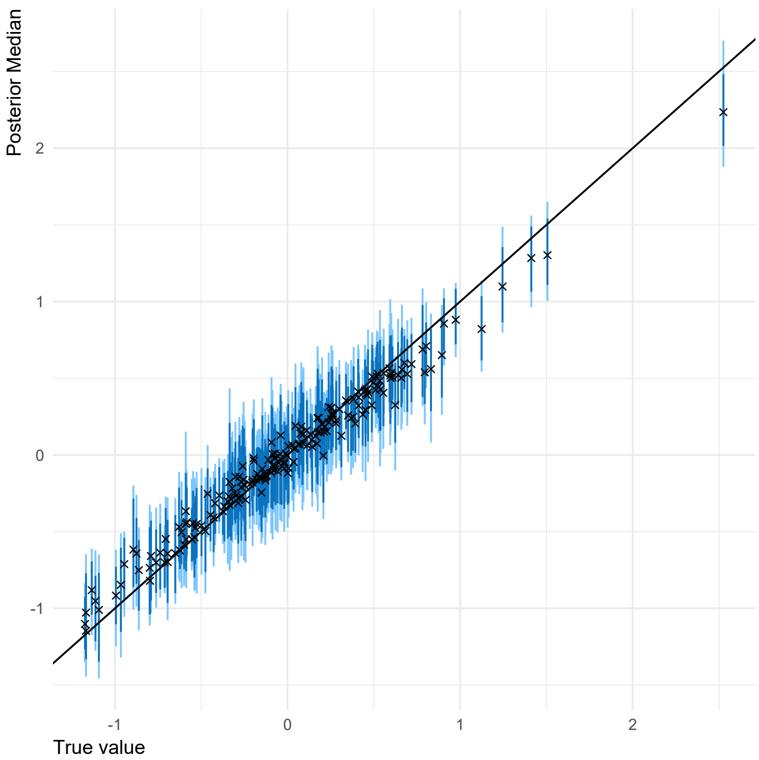

Figure 6: Simulation study results when $\boldsymbol{\theta}$ follows an ICAR distribution and the variance of the hierarchical difficulties equals one. Light blue segments represent 95% intervals for the median values, while dark blue segments represent 90% intervals. True values are shown as black crosses.

Hence, an ICAR prior can be considered when fitting the proposed model. We noticed, however, that the chains for θ_i tend to take much longer to converge when compared to the model that assumes a proper CAR prior when using the full dataset described in the paper. We ran the algorithms in `Nimble` and `Stan`. This is because they use different Markov chain Monte Carlo sampling algorithms; the goal was to check if one was more efficient than the other in attaining convergence. We ran the algorithm in `Stan` for 20,000 iterations to obtain effective sample sizes of at least 250. It took 44.5 hours to run the algorithm in `Stan`. For `Nimble`, the algorithm was run for 100,000 iterations which took a similar amount of time. Supplementary materials Section E.3 present the results obtained for the real data analysis.

E Further results of the Ghana data analysis

This section provides further details on the different fitted models. Section E.1 provides some diagnostic plots to show convergence of the chains of the main parameters of the model. Then Section E.2 provides a comparison of the posterior credible intervals of the index under different prior specifications. Finally, Section E.3 provides the results obtained from a model that assumes an intrinsic conditional autoregressive (ICAR) prior specification for the factor $\boldsymbol{\theta}$.

E.1 Diagnostic plots

This section shows the behavior of some diagnostic tools to check convergence of the MCMC algorithm used to fit the different models.

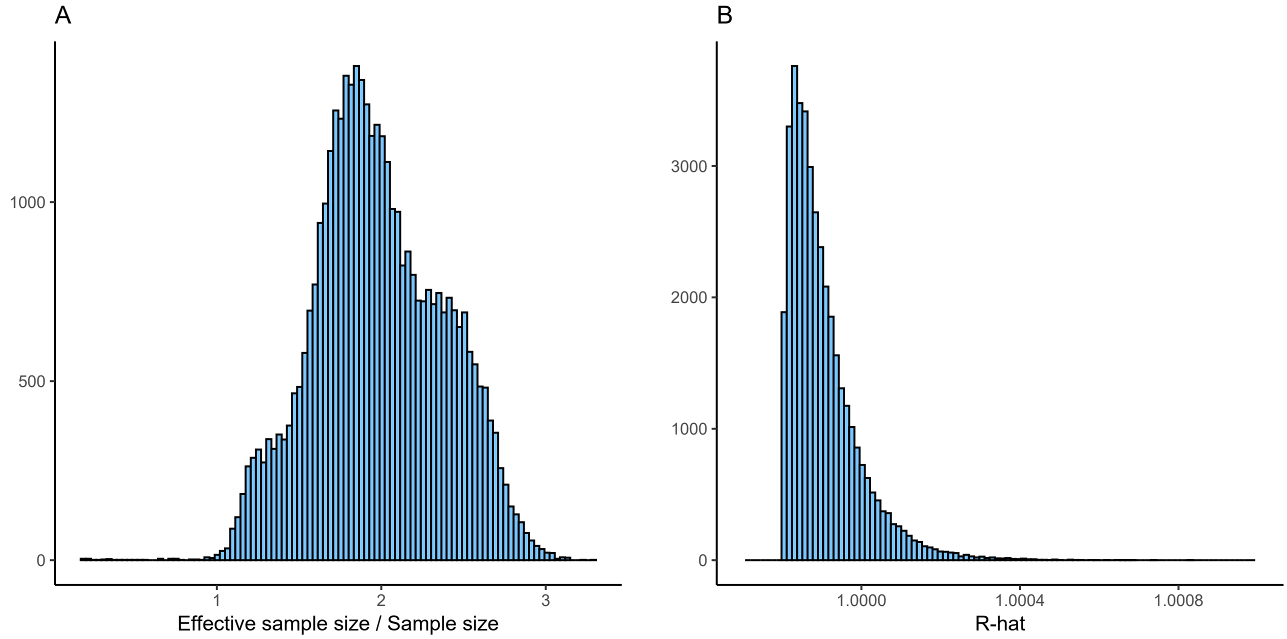

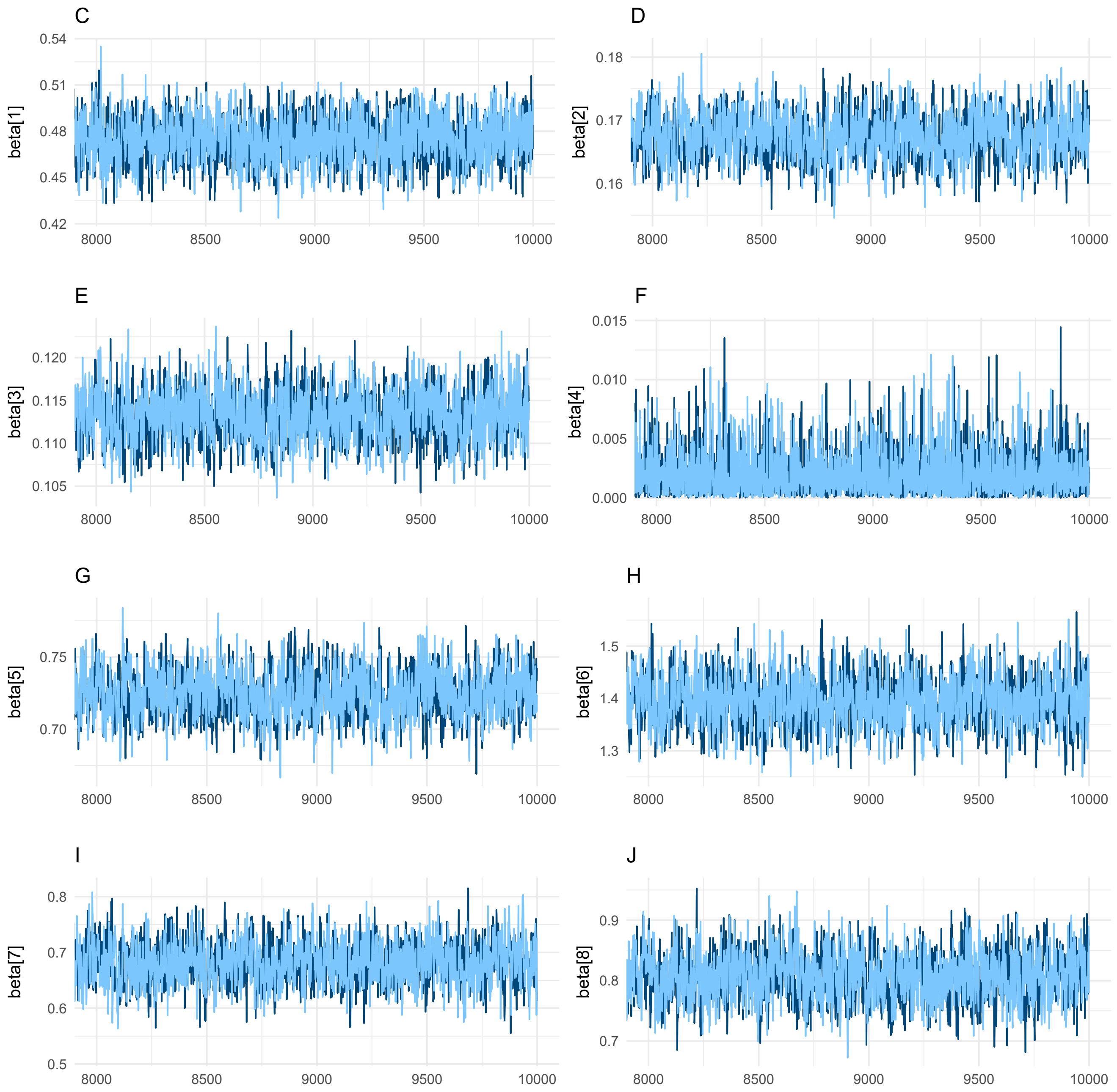
C
beta[1]
0.54
0.51
0.48
0.45
0.42
8000
8500
9000
9500
10000
D
beta[2]
0.18
0.17
0.16
8000
8500
9000
9500
10000
E
beta[3]
0.120
0.115
0.110
0.105
8000
8500
9000
9500
10000
F
beta[4]
0.015
0.010
0.005
0.000
8000
8500
9000
9500
10000
G
beta[5]
0.75
0.70
8000
8500
9000
9500
10000
H
beta[6]
1.5
1.4
1.3
8000
8500
9000
9500
10000
I
beta[7]
0.8
0.7
0.6
0.5
8000
8500
9000
9500
10000
J
beta[8]
0.9
0.8
0.7
8000
8500
9000
9500
10000

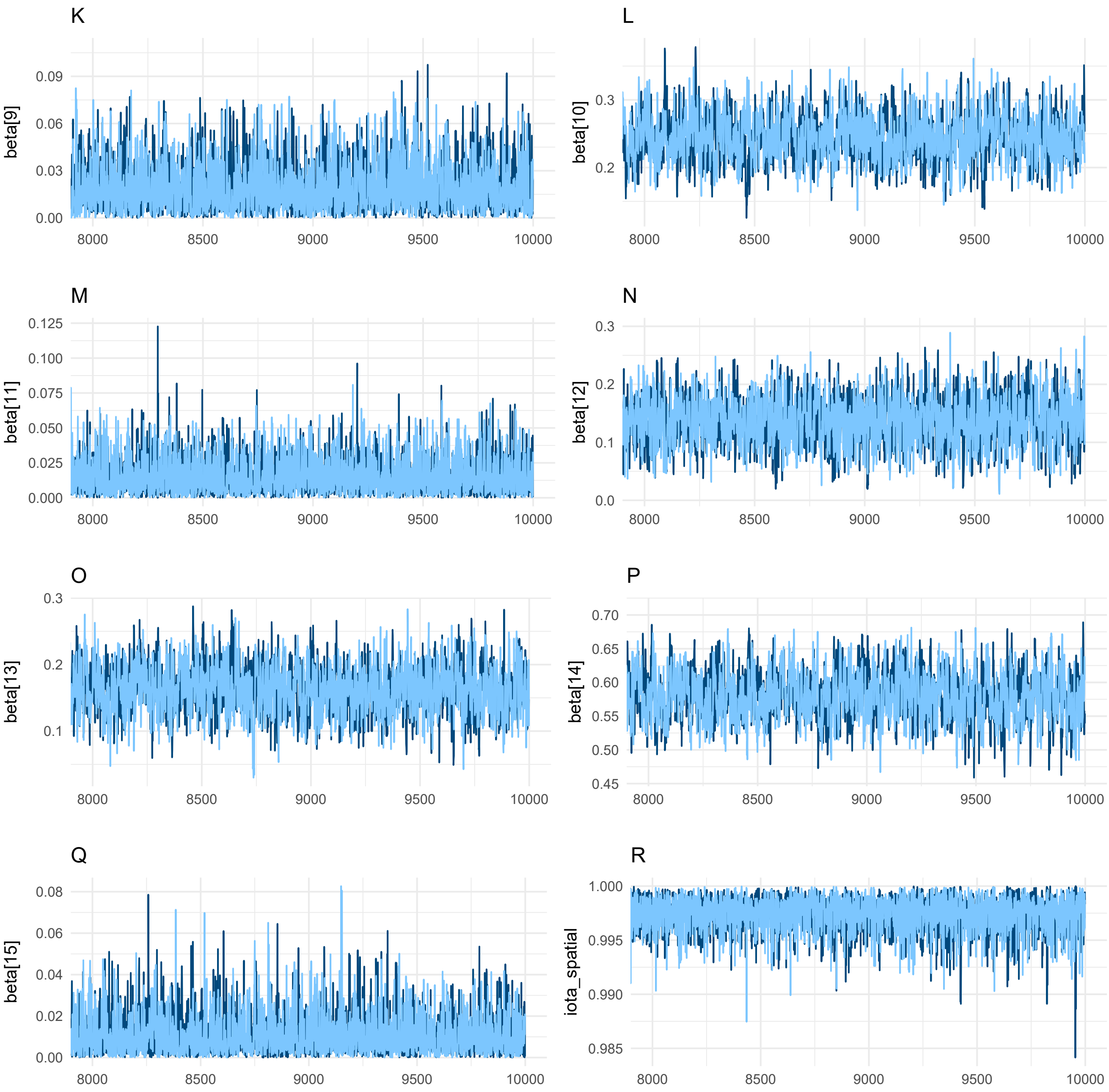
K
beta[9]
0.00
0.03
0.06
0.09
L
beta[10]
0.2
0.3
M
beta[11]
0.000
0.025
0.050
0.075
0.100
0.125
N
beta[12]
0.0
0.1
0.2
0.3
O
beta[13]
0.1
0.2
0.3
P
beta[14]
0.45
0.50
0.55
0.60
0.65
0.70
Q
beta[15]
0.00
0.02
0.04
0.06
0.08
R
iota_spatial
0.985
0.990
0.995
1.000
8000
8500
9000
9500
10000

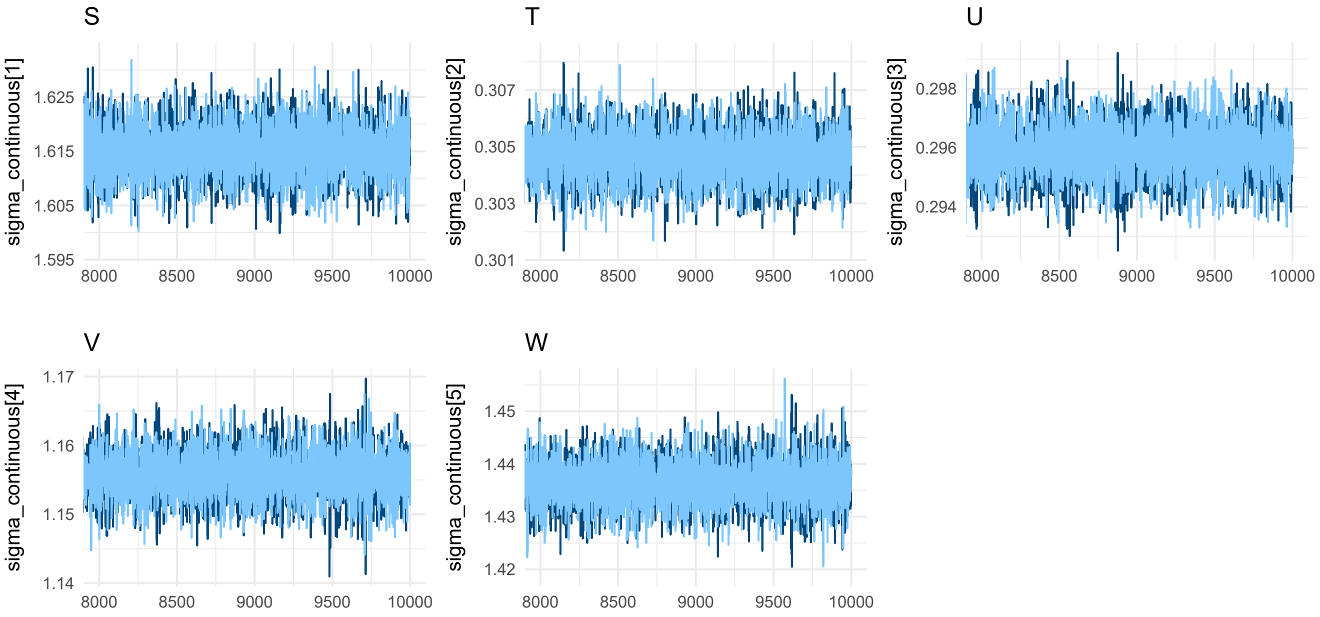
S
T
U
V
W
sigma_continuous[1]
sigma_continuous[2]
sigma_continuous[3]
sigma_continuous[4]
sigma_continuous[5]
1.625
1.615
1.605
1.595
0.307
0.305
0.303
0.301
0.298
0.296
0.294
1.17
1.16
1.15
1.14
1.45
1.44
1.43
1.42
8000
8500
9000
9500
10000

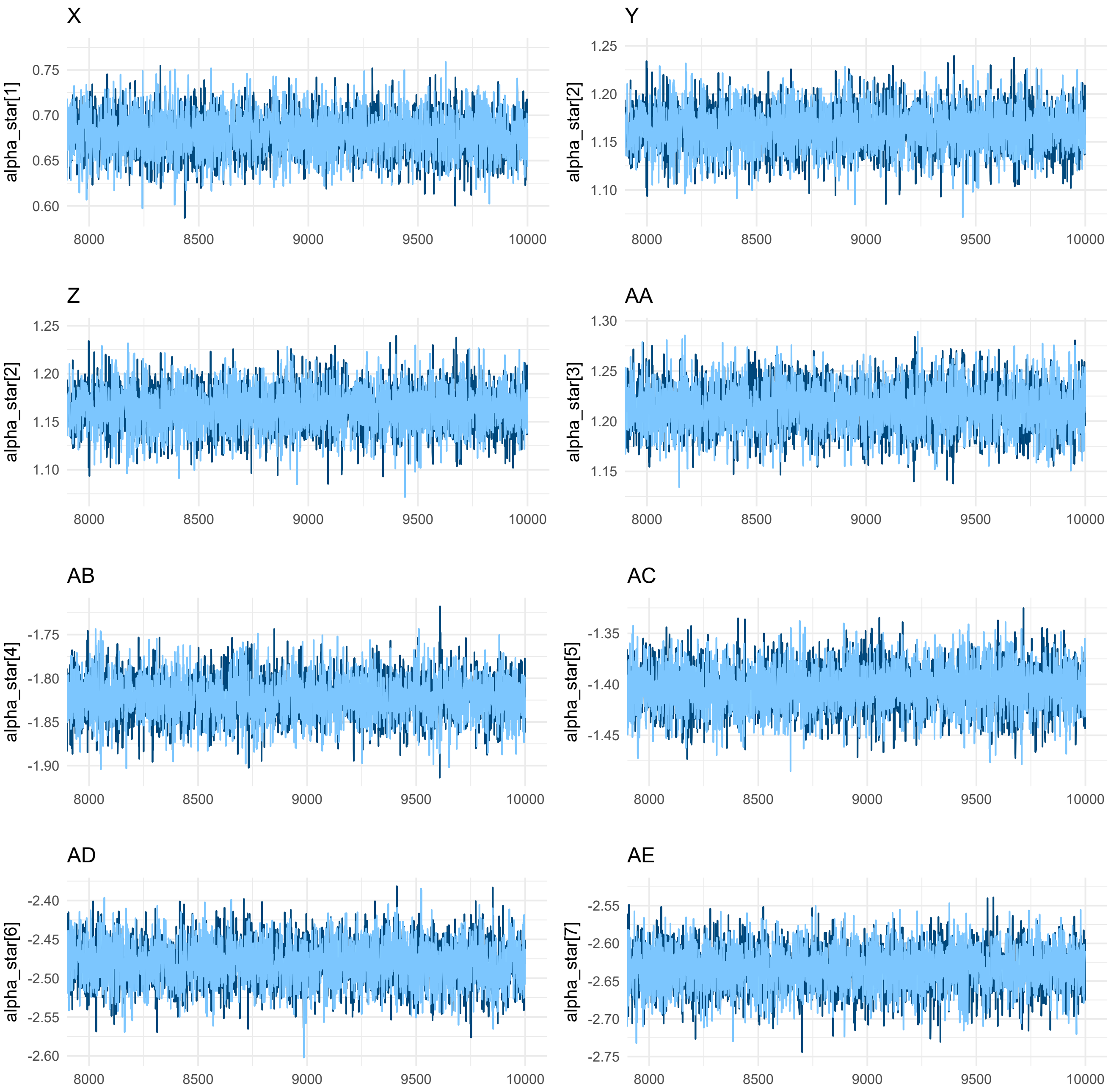
X
alpha_star[1]
0.60
0.65
0.70
0.75
8000
8500
9000
9500
10000
Y
alpha_star[2]
1.10
1.15
1.20
1.25
Z
alpha_star[2]
1.10
1.15
1.20
1.25
AA
alpha_star[3]
1.15
1.20
1.25
1.30
AB
alpha_star[4]
-1.90
-1.85
-1.80
-1.75
AC
alpha_star[5]
-1.45
-1.40
-1.35
AD
alpha_star[6]
-2.60
-2.55
-2.50
-2.45
-2.40
AE
alpha_star[7]
-2.75
-2.70
-2.65
-2.60
-2.55

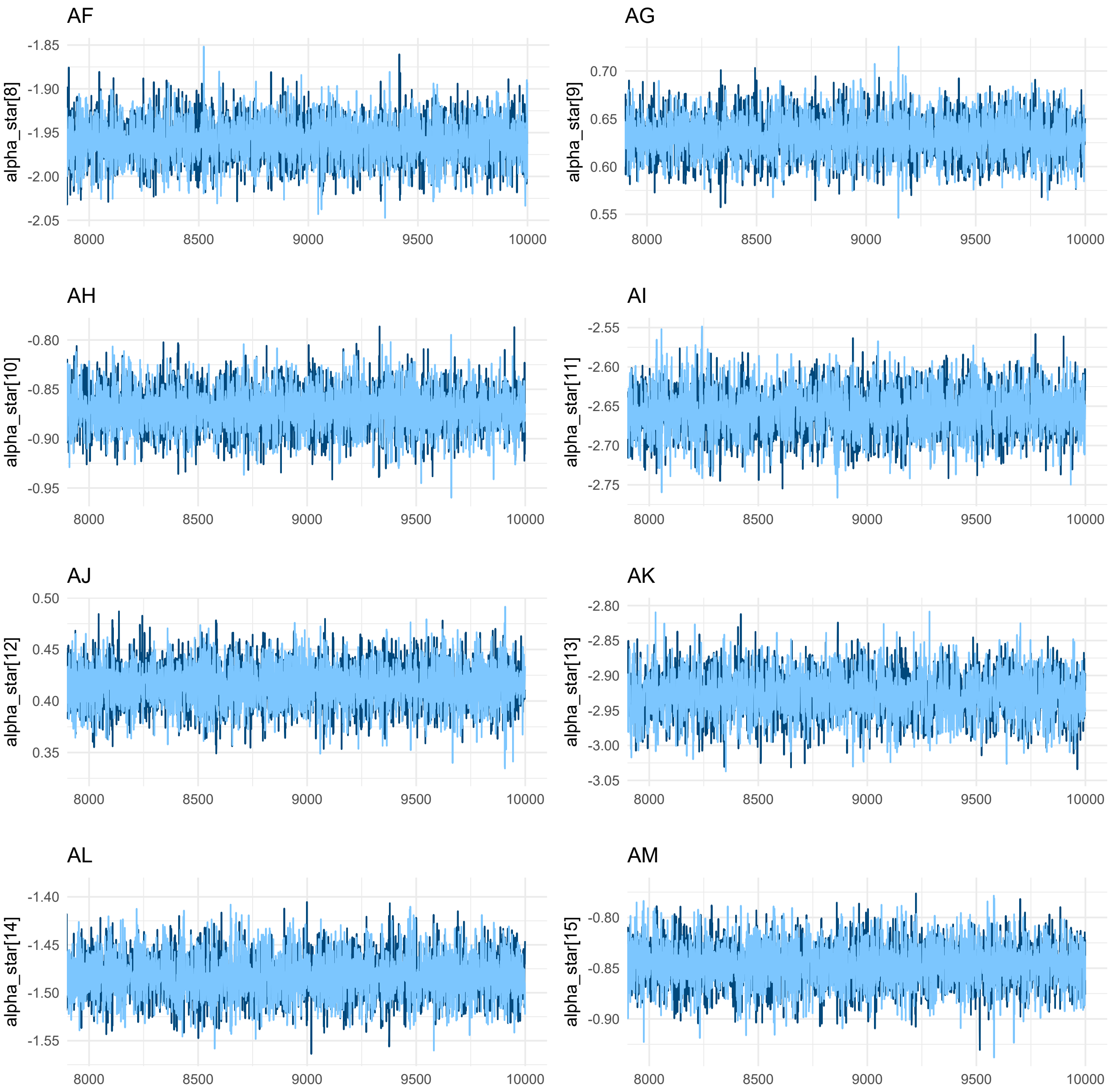
AF
alpha_star[8]
AG
alpha_star[9]
AH
alpha_star[10]
AI
alpha_star[11]
AJ
alpha_star[12]
AK
alpha_star[13]
AL
alpha_star[14]
AM
alpha_star[15]
8000
8500
9000
9500
10000

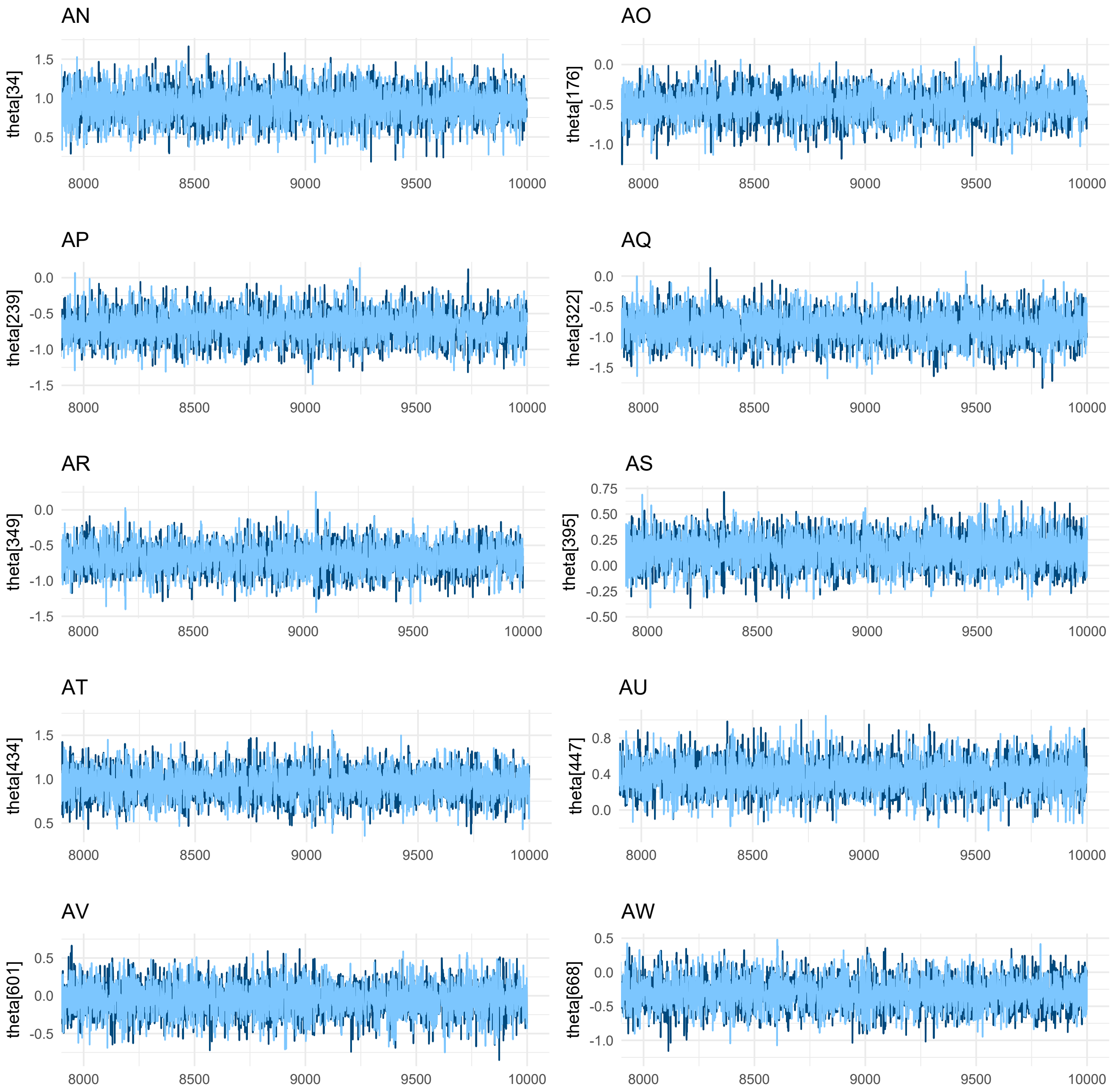
AN
theta[34]
AO
theta[176]
AP
theta[239]
AQ
theta[322]
AR
theta[349]
AS
theta[395]
AT
theta[434]
AU
theta[447]
AV
theta[601]
AW
theta[668]
8000
8500
9000
9500
10000

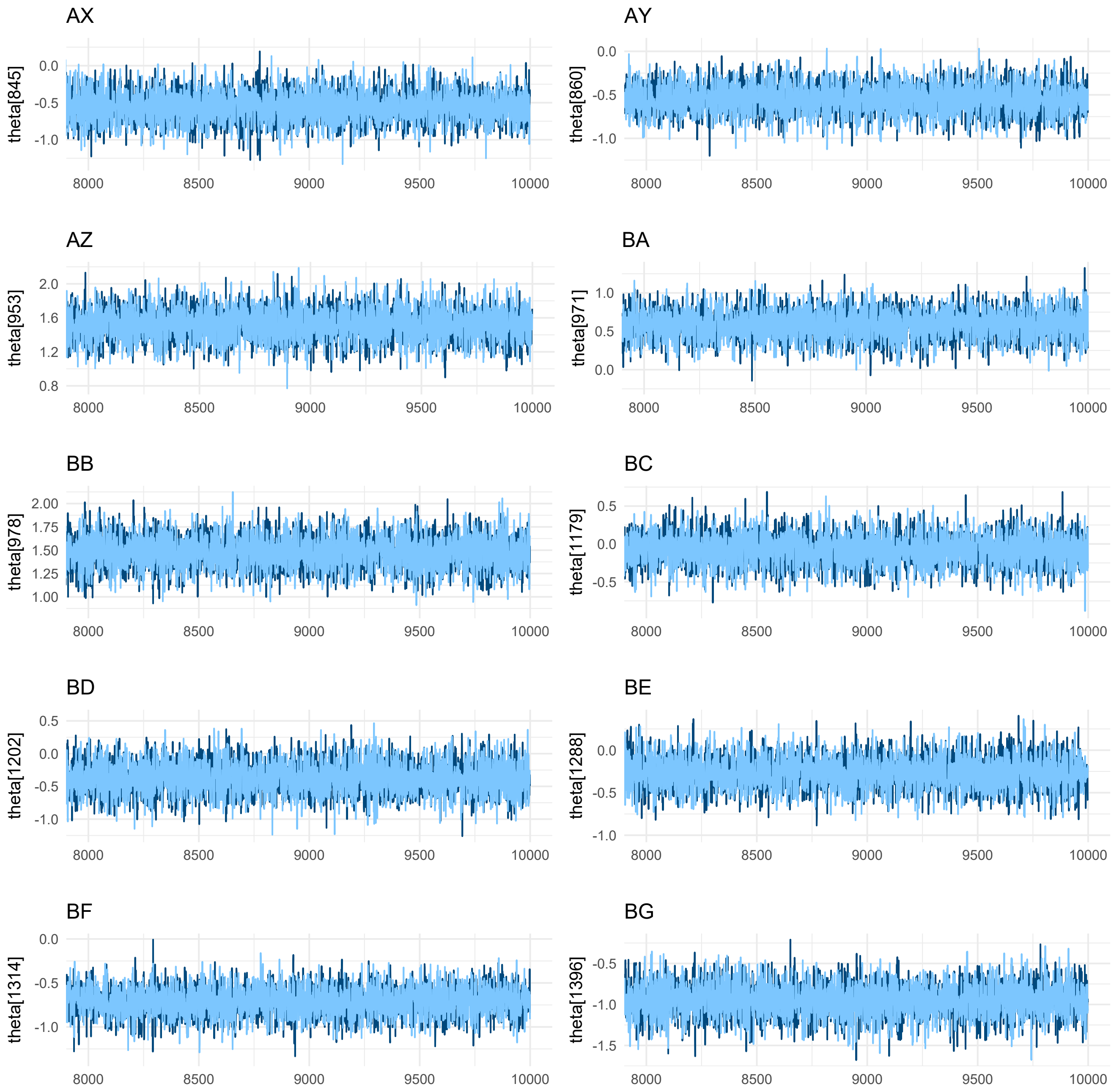
AX
theta[845]
0.0
-0.5
-1.0
AY
theta[860]
0.0
-0.5
-1.0
AZ
theta[953]
2.0
1.6
1.2
0.8
BA
theta[971]
1.0
0.5
0.0
BB
theta[978]
2.00
1.75
1.50
1.25
1.00
BC
theta[1179]
0.5
0.0
-0.5
BD
theta[1202]
0.5
0.0
-0.5
-1.0
BE
theta[1288]
0.0
-0.5
-1.0
BF
theta[1314]
0.0
-0.5
-1.0
BG
theta[1396]
-0.5
-1.0
-1.5
8000
8500
9000
9500
10000

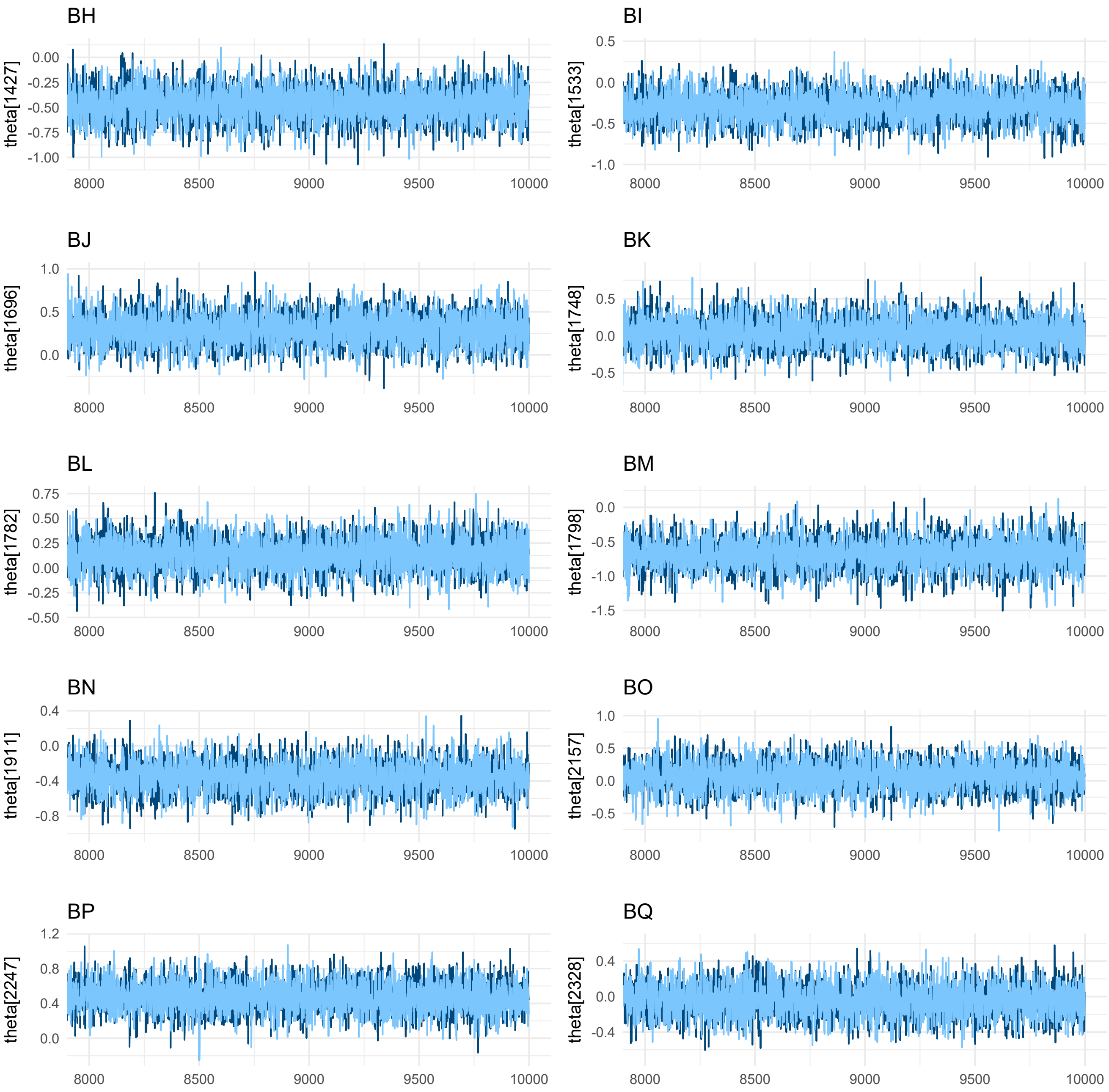

Figure 7: Panel A shows the distribution of the effective sample size and panel B the R-hat of all the parameters in the model. Panels C through AM depict trace plots of the parameters in the model. Panels AN through BQ show trace plots of the indices of 30/2424 randomly drawn areas.

E.2 Comparison of the estimated index under the different fitted models

Panels of Figure 8 show the posterior summaries of the index θ_i across the 2,424 enumeration areas under the different fitted models. We note that using a proper CAR prior for $\boldsymbol{\theta}$ (in comparison to assuming prior independence) greatly improved the precision around estimates. The same can be said of the hierarchical prior for $\boldsymbol{\alpha}$. The model benefited from borrowing strength across the map from the spatial structure noted in the variables in our exploratory analysis.

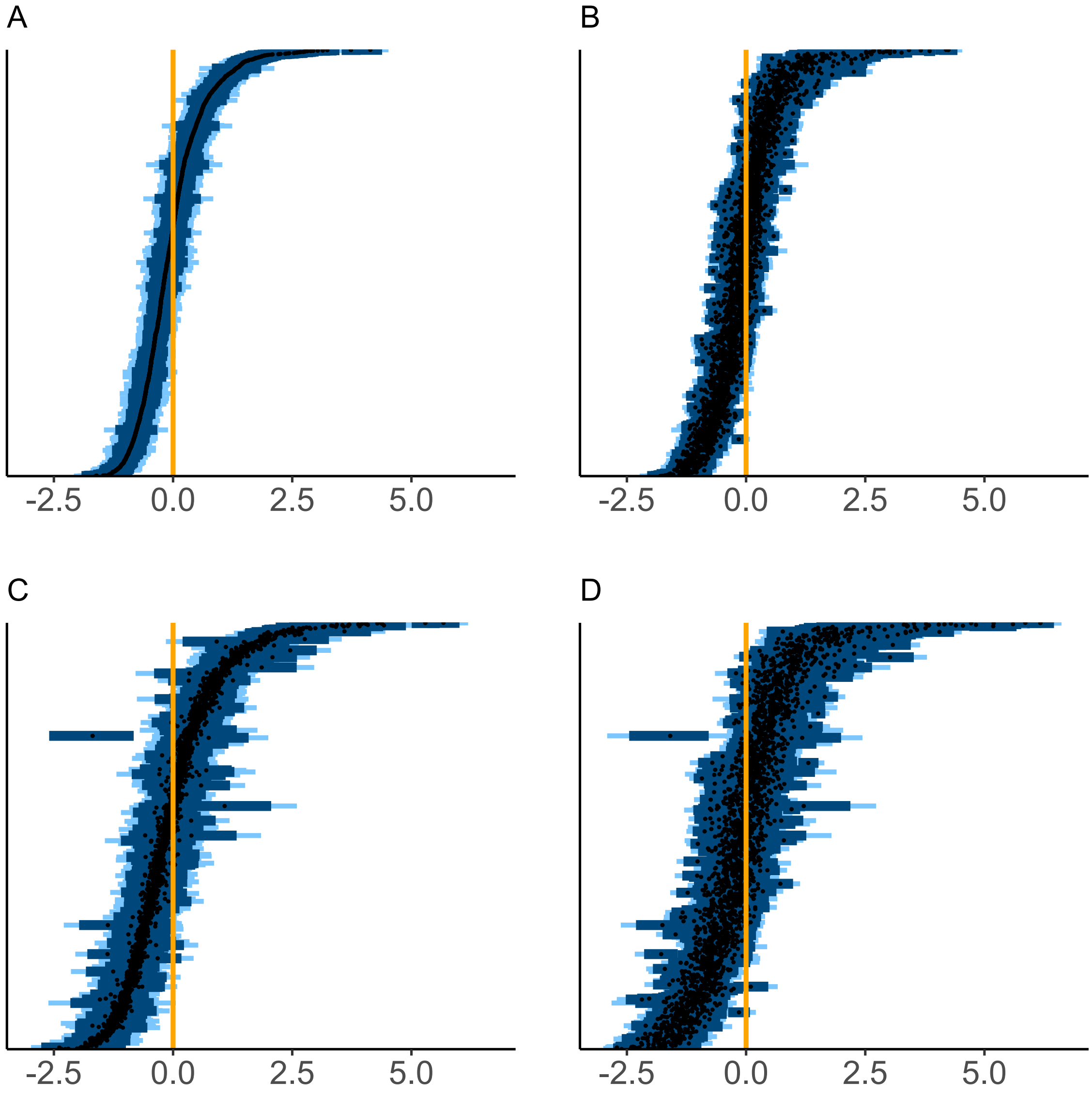

Figure 8: Posterior 80% (dark blue) and 95% (light blue) credible intervals for all four fitted models to the Ghana data. Panels A and B impose a CAR prior to $\boldsymbol{\theta}$ while C and D impose a normal prior. Panels A and C impose a hierarchical structure to $\boldsymbol{\alpha}$. Each of them is ordered according to the ordering of the posterior medians for the chosen model, in the left-top sub-figure.

Panels of Figure 9 provide pairwise scatter plots of the posterior means of the estimated index, $\boldsymbol{\theta}$, under the different prior specifications for $\boldsymbol{\alpha}$. In both cases of prior for $\boldsymbol{\alpha}$, the estimated θ_i's are highly correlated. However, they are not equal as shown by the $y = x$ yellow line. Even though the relative ordering of their discrimination parameters $\boldsymbol{\beta}$ are

conserved, their scale differs, suggesting a different combination identified for $\boldsymbol{\beta\theta}$.

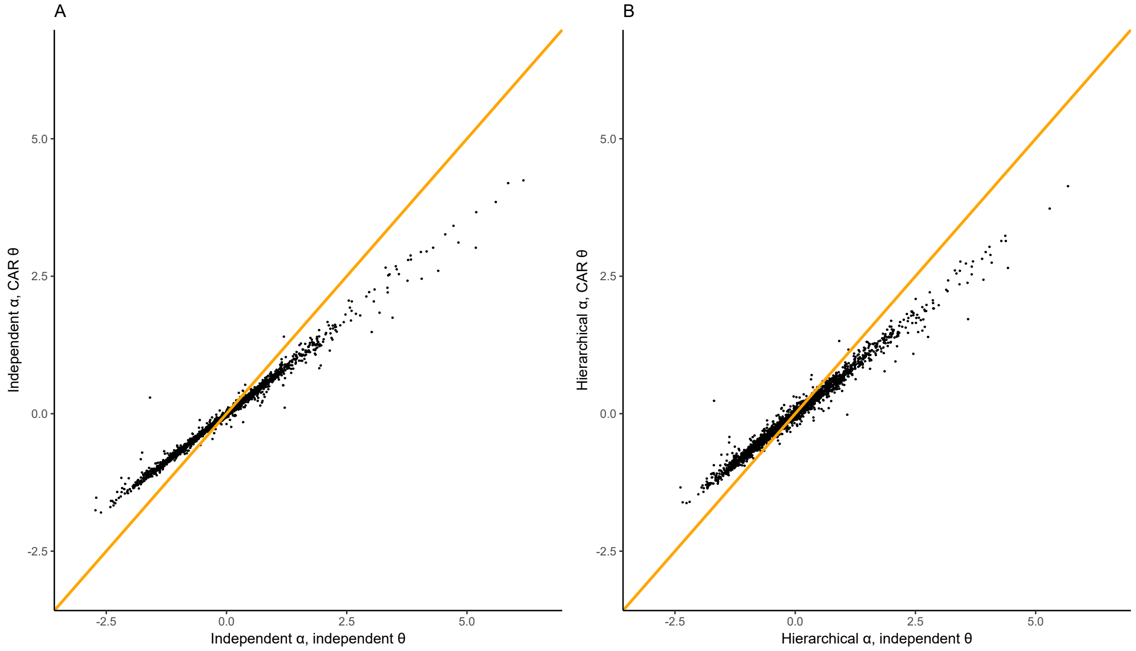

Figure 9: Scatter plots of the posterior estimates of the indices $\boldsymbol{\theta}$ when they are given an independent or spatial prior. Panel B imposes a hierarchical structure to $\boldsymbol{\alpha}$ while panel A does not.

E.3 Results from a model that assumes an ICAR prior for the index θ_i

As pointed out by one of the reviewers, the correlation parameter, ι_θ, of the proper CAR prior for the factor (see Section 2.1) is estimated very close to 1, suggesting that an ICAR prior could be considered for the indices $\boldsymbol{\theta}$. The ICAR is an improper prior distribution, and a sum-to-zero constraint can be imposed to guarantee a proper posterior distribution.

It should be noted that the chains leading to the results shown in panels of Figure 10 showed some convergence issues. Even though the model was run for double the amount of iterations of the proper CAR model, the effective sample sizes (ESS) of some indices (θ) and discrimination parameters (β) were as low as 276. Fitting 20,000 iterations of this model took 44.5 hours running the chains in parallel. The maximal value of $\hat{R}$ was 1.008. Because of the relatively small ESS, the results obtained should be interpreted with caution, especially for variables: proportion without secondary school, proportion of illiteracy, households with

no flush toilet, indoor piping, internet or computers. Panels of Figure 11 illustrate this convergence issue by showing the trace plots of two of these variables which present quite high-autocorrelated chains. The WAIC for this model was 1,179,282, which is better than the non-spatial models, but worse than the proper CAR models. For these reasons, in the main text, we focus on the results based on the proper CAR prior for $\boldsymbol{\theta}$.

Panels of Figure 10 provide the posterior summaries of some of the parameters of the model under the ICAR prior for $\boldsymbol{\theta}$. Note that they closely resemble the results obtained in the main paper (compare panels of Figure 10 with Figures 2, 3, and 4 of the main text).

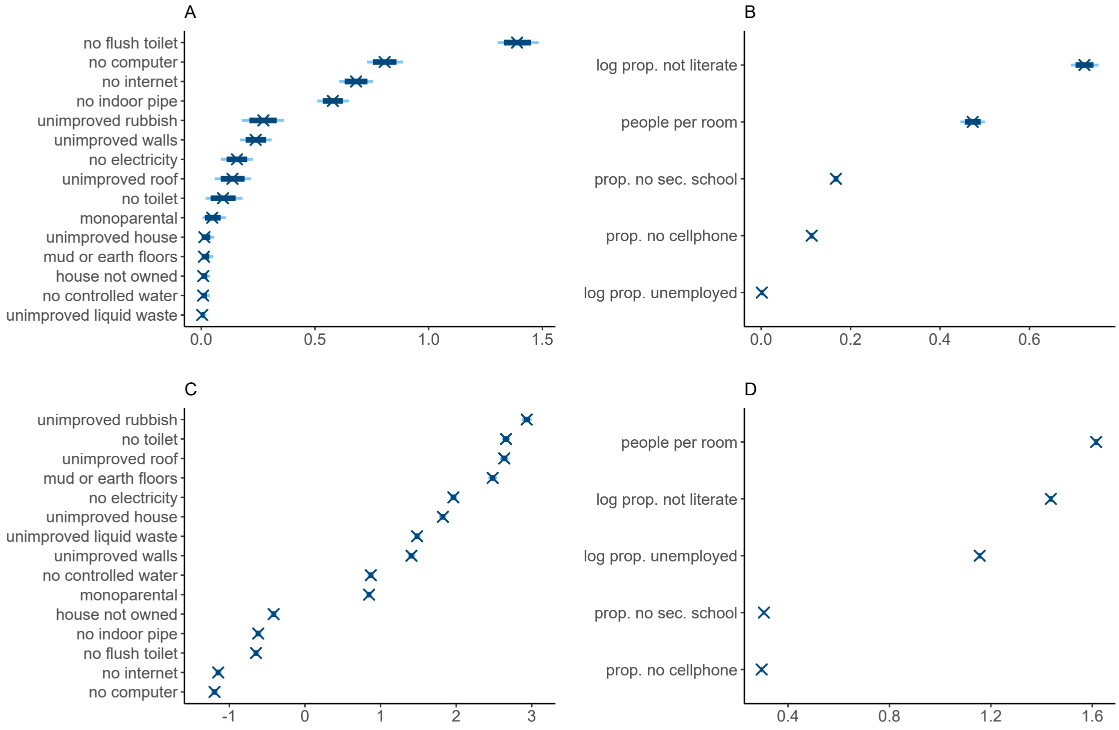

Figure 10: Posterior credible intervals with the indices following an ICAR prior imposed to $\boldsymbol{\theta}$. The results obtained are almost identical to those obtained with the proper CAR described in the paper. The relative ordering as well as the magnitudes of all parameters are similar. Panel A shows the discrimination parameters of dichotomous variables, B the discrimination parameters of continuous variables, C the negative difficulty parameters and D the standard deviations of the continuous variables.

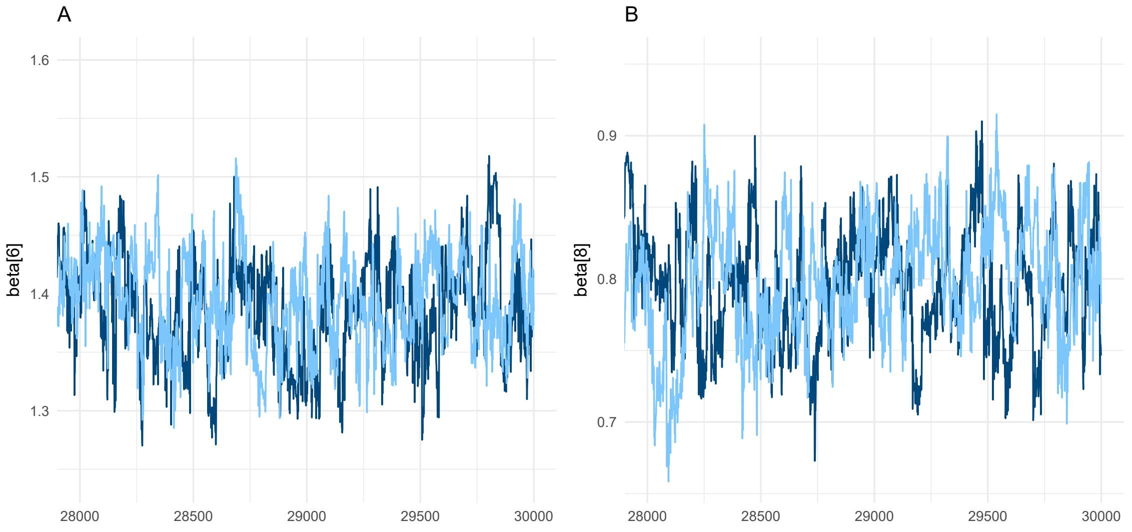

Figure 11: Trace plots of discrimination parameters with the indices following an ICAR prior

F Stan code

```
// The data consists of dichotomous and continuous entries.
// The continuous outcomes MUST BE CENTERED and come FIRST
// Model: y_ijk = alpha_ik + beta_k ' theta_i + e_ijk
// Is equivalent to a mix of IRT probit model and factor
    analysis.
// The latent factor is thought to vary amongst EAs (
    enumeration areas), but not between households within EAS.
//Theta follows an exact CAR distribution with tau set to 1.
//Alpha has a hierarchical structure.

functions {
  /**Taken from https://mc-stan.org/users/documentation/case-
      studies/mbjoseph-CARStan.html
  * Return the log probability of a proper conditional
      autoregressive (CAR) prior with a sparse representation
      for the adjacency matrix.
  *
  * @param phi Vector containing the parameters with a CAR
      prior
  * @param tau Precision parameter for the CAR prior (real)
  * @param alpha Dependence (usually spatial) parameter for
      the CAR prior (real)
  * @param W_sparse Sparse representation of adjacency matrix
      (int array)
```

```
* @param n Length of phi (int)
* @param W_n Number of adjacent pairs (int)
* @param D_sparse Number of neighbours for each location (
  vector)
* @param lambda Eigenvalues of D^{-1/2}*W*D^{-1/2} (vector)
*
* @return Log probability density of CAR prior up to
  additive constant
*/
real sparse_car_lpdf(vector phi, real tau, real alpha,
  int[,] W_sparse, vector D_sparse, vector lambda, int n,
    int W_n) {
  row_vector[n] phit_D; // phi' * D
  row_vector[n] phit_W; // phi' * W
  vector[n] ldet_terms;

  phit_D = (phi .* D_sparse)';
  phit_W = rep_row_vector(0, n);
  for (i in 1:W_n) {
    phit_W[W_sparse[i, 1]] = phit_W[W_sparse[i, 1]] + phi[
      W_sparse[i, 2]];
    phit_W[W_sparse[i, 2]] = phit_W[W_sparse[i, 2]] + phi[
      W_sparse[i, 1]];
  }

  for (i in 1:n) ldet_terms[i] = log1m(alpha * lambda[i]);
  return 0.5 * (n * log(tau)
```

```
                    + sum(ldet_terms)
                    - tau * (phit_D * phi - alpha * (phit_W *
                      phi)));
  }
}

data {
  int <lower=1> nEa ; //number of EAS
  int <lower=1> nObs ; //number of observations (households)
  int <lower=1> nVar ; //number of variables observed
  int <lower=1> nContinuous ; //number of continuous variables
      observed
  int <lower=1, upper=nEa> ea[nObs]; //EA for observation n
  real x_cont[nObs,nContinuous]; //observed continuous
     variables
  int <lower=0,upper=1> x_dichot[nObs,nVar-nContinuous]; //
     observed dichotomous variables

  matrix <lower = 0, upper = 1> [nEa, nEa] W; //adjencency
     matrix
  matrix <lower = 0> [nEa, nEa] D; //diagonal matrix with the
     number of neighbours of each EA
  int <lower = 0> W_n; // number of adjacent region pairs
}

transformed data {
  int W_sparse[W_n, 2]; // adjacency pairs
```

```
vector[nEa] D_sparse; // diagonal of D (number of neighbours
    for each area)
vector[nEa] lambda;   // eigenvalues of invsqrtD * W *
   invsqrtD

{ // generate sparse representation for W
int counter;
counter = 1;
// loop over upper triangular part of W to identify
   neighbour pairs
  for (i in 1:(nEa - 1)) {
    for (j in (i + 1):nEa) {
      if (W[i, j] == 1) {
        W_sparse[counter, 1] = i;
        W_sparse[counter, 2] = j;
        counter = counter + 1;
      }
    }
  }
}
for (i in 1:nEa) D_sparse[i] = sum(W[i]);
{
  vector[nEa] invsqrtD;
  for (i in 1:nEa) {
    invsqrtD[i] = 1 / sqrt(D_sparse[i]);
  }
```

```
    lambda = eigenvalues_sym(quad_form(W, diag_matrix(invsqrtD
        )));
  }
}

parameters{
  matrix[nEa,nVar-nContinuous] alpha; //difficulty parameters,
      unique to EAs
  vector<lower=0> [nVar] beta; //discrimination parameters
  vector[nEa] theta; //factor scores for each EA
  vector <lower=0> [nContinuous] sigma_continuous; //standard
     deviations for continuous variables
  vector[nVar-nContinuous] alpha_star; //overall mean
     difficulty
  real<lower = 0, upper = 1> iota_spatial; //controls spatial
     dependence
}

model {
  matrix[nObs,nVar-nContinuous] x_star_dichot; // continuous
     latent form of the dichotomous observations
  matrix[nObs,nContinuous] x_star_cont;// continuous latent
     form of the continuous observations
  matrix[nObs,nVar] Pi; //matrix of probabilities for the
     observed dichotomous data
```

```
theta ~ sparse_car(1, iota_spatial, W_sparse, D_sparse,
   lambda, nEa, W_n);
//iota has a uniform 0-1 prior
alpha_star ~ std_normal();
beta ~ std_normal();
sigma_continuous~cauchy(0,1);

for (i in 1:nEa){
  for (j in 1:(nVar-nContinuous)) alpha[i,j]~normal(
     alpha_star[j],1);
}

for (n in 1:nObs){
  for (k in 1:nContinuous){
    x_star_cont[n,k] = beta[k]*theta[ea[n]];
    x_cont[n,k] ~ normal(x_star_cont[n,k], sigma_continuous[
       k]);
  }
  for (k in (nContinuous+1):nVar){
    x_star_dichot[n,k-nContinuous] = alpha[ea[n],k-
       nContinuous] + beta[k]*theta[ea[n]];
    Pi[n,k-nContinuous] = Phi(x_star_dichot[n,k-nContinuous
       ]);
    x_dichot[n,k-nContinuous] ~ bernoulli(Pi[n,k-nContinuous
       ]);
  }
}
```

}

References

De Oliveira, V. (2000). Bayesian prediction of clipped gaussian random fields. *Computational Statistics & Data Analysis*, 34 (3), 299–314.

Higgs, M. D. and Hoeting, J. A. (2010). A clipped latent variable model for spatially correlated ordered categorical data. *Computational Statistics & Data Analysis*, 54 (8), 1999–2011.

Rue, H. and Held, L. (2005). *Gaussian Markov random fields: theory and applications.* : Chapman and Hall/CRC.

Schliep, E. M. and Hoeting, J. A. (2013). Multilevel latent gaussian process model for mixed discrete and continuous multivariate response data. *Journal of Agricultural, Biological, and Environmental Statistics*, 18, 492–513.

Wang, F. and Wall, M. M. (2003). Generalized common spatial factor model. *Biostatistics*, 4 (4), 569–582.
